# A comparative analysis of the nervous system of cheilostome bryozoans

**DOI:** 10.1186/s40850-021-00084-8

**Published:** 2021-06-11

**Authors:** Jakob Prömer, Andy Sombke, Thomas Schwaha

**Affiliations:** Department of Evolutionary Biology, University of Vienna, Althanstraße 14, 1090 Vienna, Austria

**Keywords:** Neuroanatomy, Lophotrochozoa, Gymnolaemata, Myolaemata, Alpha tubulin staining

## Abstract

**Background:**

Bryozoans are sessile aquatic suspension feeders in mainly marine, but also freshwater habitats. Most species belong to the marine and calcified Cheilostomata. Since this taxon remains mostly unstudied regarding its neuroanatomy, the focus of this study is on the characterization and ground pattern reconstruction of the autozooidal nervous system based on six representatives.

**Results:**

A common neuronal innervation pattern is present in the investigated species: a cerebral ganglion is located at the base of the lophophore, from where neurite bundles embrace the mouth opening to form a circumoral nerve ring. Four neurite bundles project from the cerebral ganglion to innervate peripheral areas, such as the body wall and parietal muscles via the tentacle sheath. Five neurite bundles comprise the main innervation of the visceral tract. Four neurite bundles innervate each tentacle via the circumoral nerve ring. Mediofrontal tentacle neurite bundles emerge directly from the nerve ring. Two laterofrontal- and one abfrontal tentacle neurite bundles emanate from radial neurite bundles, which originate from the cerebral ganglion and circumoral nerve ring in between two adjacent tentacles. The radial neurite bundles terminate in intertentacular pits and give rise to one abfrontal neurite bundle at the oral side and two abfrontal neurite bundles at the anal side. Similar patterns are described in ctenostome bryozoans.

**Conclusions:**

The present results thus represent the gymnolaemate situation. Innervation of the tentacle sheath and visceral tract by fewer neurite bundles and tentacular innervation by four to six tentacle neurite bundles support cyclostomes as sister taxon to gymnolaemates. Phylactolaemates feature fewer distinct neurite bundles in visceral- and tentacle sheath innervation, which always split in nervous plexus, and their tentacles have six neurite bundles. Thus, this study supports phylactolaemates as sistergroup to myolaemates.

## Background

Bryozoans are a group of colonial, aquatic suspension-feeders consisting of over 6.000 recent and over 15.000 fossil described species [1]. Along with other phyla such as mollusks, nemerteans or phoronids, they belong to the protostome Lophotrochozoa, and lately are reunited with phoronids and brachiopods as monophyletic lophophorates (e.g. [2, 3]).

All bryozoan colonies consist of modules or individuals termed zooids that through iterative budding processes from a found zooid (in sexual development the ancestrula) create colonies of sometimes extensive sizes. Zooids are traditionally, but artificially, divided into a cystid, which essentially is the body wall, and the polypide that contains all major organ systems such as the ciliated tentacle crown, the lophophore, and the u-shaped digestive tract [4].

Two distinct taxa are distinguishable among bryozoans: Phylactolaemata and Myolaemata. The former is a small group of freshwater bryozoans, whereas the latter is a large bulk of mostly marine bryozoans [5]. Myolaemates can be divided into the Stenolaemata with the single recent clade Cyclostomata and the Gymnolaemata, comprising the paraphyletic “Ctenostomata” and the monophyletic Cheilostomata [6, 7]. Cyclostomes and cheilostomes have mineralized skeletons and are the largest, dominant groups of bryozoans, comprising around 800 and over 5.000 species, respectively [7]. Stenolaemates were dominant in the Paleozoic, whereas cheilostomes originated from the Mesozoic and immensely started to thrive during that period [8, 9].

Cheilostome success and diversity was most likely triggered by the evolution of brooding structures and lecithotrophic larvae [10, 11], but also a series of other characters. The latter include rapid colonial growth [12, 13], large lophophores and feeding currents [14], polymorphism [15] and colonial integration [see 5]. Colonial integration seems to be highly influenced by the presence of a complex communication system of so-called funicular strands that interconnect individual zooids of the colony [5]. The nervous system, however, is little investigated in cheilostome bryozoans. A series of studies employing more state-of-the-art techniques such as immunocytochemistry in combination with confocal laser scanning microscopy were recently conducted on numerous bryozoans including larvae [16–19], phylactolaemates [20–22], cyclostomes [23–25] and ctenostomes [26–29]. The large clade of Cheilostomata remains mostly unstudied and our knowledge on their nervous system mainly relies on older silver and methylene blue stainings predominantly on the membraniporine/malacostegan genus *Electra* [see 4, 5]. Only few studies were conducted on neurotransmitter distribution such as serotonin and FMRF-amide [30, 31], which, however, are restricted to few neuronal elements of the lophophoral base and fail to show a complete picture of the nervous system [31].

Owing to the lack of more complete modern investigations on the cheilostome nervous systems, but also its variation among different taxa, the aim of this study is to analyze the nervous system of cheilostome bryozoans on a broader scale including several taxonomic high-level groups and modern methods. More specifically, older results will be compared methodologically, and variation and ground pattern of the cheilostome nervous system are specific foci of this study.

## Methods

### Sampling, identification and fixation

**Fig. 1:**
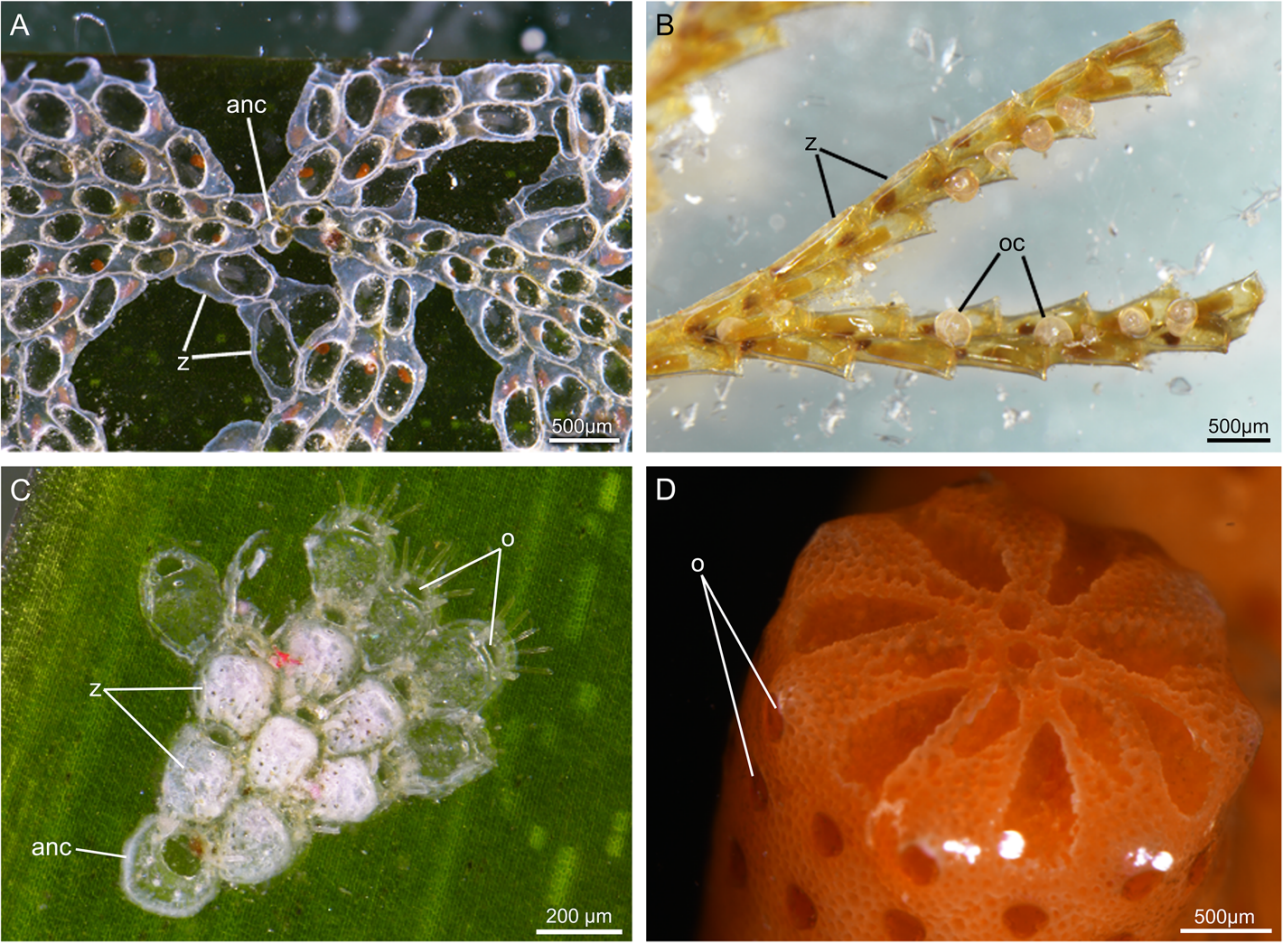
Colony morphology of few cheilostome bryozoan species. A *Electra posidoniae* encrusting colony on *Posidonia oceanica*. B *Bugula neritina* erect dichotomous branching colony with biserial branches. C *Fenestrulina joannae* encrusting colony on *P. oceanica*. D *Myriapora truncata* thick massive radial branch of irregularly branching colony. Abbreviations: anc – ancestrula, o – operculum, oc – ovicell, z – zooid

Six species from four major cheilostome taxa were analyzed: *Bugula neritina*, *Chorizopora brongniartii*, *Collarina balzaci*, *Electra posidoniae*, *Fenestrulina joannae*, *Myriapora truncata* (see Fig. 1) were sampled from different substrate types at Valsaline Bay, Pula, in Istria, Croatia (44°51′59″ N, 13°50′01″ E) in May and June 2019 and identified using [32]. Identification was documented with a Nikon SMZ25 stereomicroscope equipped with a DS-Ri2 microscope camera (Nikon, Chiyoda, Tokyo, Japan). The general bauplan and neuronal structures were first analyzed and evaluated in *E. posidoniae* in about 18 stained colony pieces. Approximately 3–4 colonies of the other species were stained and analyzed. Likewise, 3–4 representative and well-stained zooids were used for detailed scans.

### Immunocytochemistry

Samples were fixed in 4% paraformaldehyde (PFA) in 0.1 M phosphate buffer with pH of 7,4 (PB) for 1 h. After washing them three times for 1 h each in PBS they were stored in PB with 0.01% sodium azide. Samples were incubated in 20% ethylenediaminetetraacetic acid (EDTA) in distilled water for 1 h or 24 h depending on their calcification state prior to staining.

Decalcified samples of all six species were washed two times 20 min each in PB and incubated in a blocking solution of 6% normal goat serum (Thermo Fisher Scientific, Waltham, MA, USA), 2% dimethyl sulfoxide (DMSO) and 2% Triton X-100 in PB overnight. They were incubated with a monoclonal antibody against acetylated alpha-tubulin raised in mouse (T6793, Sigma Aldrich, St. Louis, MO, US) diluted 1:600 in PB with 2% DMSO and 2% Triton X-100 overnight. After four washes with PB 20 min each, they were incubated in goat anti mouse Alexa Fluor 568 (A11004, Thermo Fisher Scientific, Waltham, MA, USA) in 1:300 dilution together with DAPI (D1306, Thermo Fisher Scientific) in 1:100 dilution and Alexa Fluor 488 Phalloidin (A12379, Thermo Fisher Scientific) in 1:40 dilution with 2% DMSO and 2% Triton X-100 in PBS overnight. Samples were washed four times 20 min each with PBS and mounted in Fluoromount G (Southern Biotech, Birmingham, AL, USA). They were kept at 4 °C overnight for hardening of the mounting medium and subsequently analyzed using a Leica SP5 II CLSM (Leica Microsystems, Wetzlar, Germany).

### Terminology and data analysis

Characters for evaluation of the nervous system were defined using terminologies of [4, 5, 33, 34]. The center of the nervous system is referred to as cerebral ganglion, based on most recent terminology [4, 33]. All image stacks were analyzed using Amira, version 6.4 (FEI, Hillsboro, Oregon, USA). Morphological characters were evaluated as nervous system character matrix (Table. 1). Antibody stainings require opening of the cystid walls to ensure permeability. Particularly in larger species, dissection often destroyed or distorted several characters. A ground pattern analysis was performed based on the matrix and data from previous studies (Table. 2). “Ctenostomata” are the outgroup in this study. Three character states were defined for the analysis: absent (1), present (0), not applicable (−).

**Table 1:** Nervous system character matrix of the six investigated species. From the 27 defined characters, 23 were used in the ground pattern analysis. Two characters states were used in this matrix: present (1), absent (0) and (−) inapplicable

	*Bugula neritina*	*Chorizopora brongniartii*	*Collarina balzaci*	*Electra posidoniae*	*Fenestrulina joannae*	*Myriapora truncata*
**lophophoral base**						
cerebral ganglion/brain	1	1	1	1	1	1
circumoral nerve ring	1	1	1	1	1	1
trifid nerve	1	1	1	1	1	1
descending branch	1	1	1	1	0	1
annular branch	1	0	0	1	0	1
direct nerve origin sites	0	0	0	0	0	0
direct tentacle sheath nerve	1	1	1	1	1	1
**lophophore**						
radial nerves	1	1	1	1	1	1
abfrontal tentacle nerve	1	1	1	1	1	1
medio-frontal tentacle rootlets	1	1	1	1	1	1
medio-frontal tentacle nerve	1	1	1	1	1	1
latero-frontal tentacle nerve	1	1	1	1	1	1
intertentacular pits	1	1	1	1	1	1
intertentacular neurites	1	1	1	1	1	1
**visceral innervation**						
medio-visceral nerve	1	1	1	1	1	1
latero-visceral nerves	1	1	1	1	1	1
medio-latero-visceral nerve	1	0	0	1	0	1
pharyngeal plexus	1	0	1	1	1	1
visceral nerve origin site	0	0	0	0	0	0
**peripheral innervation**						
compound tentacle sheath nerve	1	1	1	1	1	1
parietal branching site	1	1	1	1	1	1
parietal muscle nerve	1	1	1	1	1	0
opercular branch	–	1	1	1	1	1
sphincter branch	1	1	1	1	1	1
parietodiaphragmatic branch	1	1	1	1	1	1
**remainder**						
interzooidal pores	0	0	0	1	0	0

**Table 2:** Nervous system character matrix including published data. Character states used in this matrix: present (1), absent (0) and missing data (?)

Author	Temereva & Kosevich (2016)	Marcus (1926)	Graupner (1930)	Schwaha et al. (2011)	Proets et al. (2019)	Weber et al. (2011)
	*Amathia gracilis*	*Farrella repens*	*Flustrellidra hispida*	*Hislopia malayensis*	*Hypophorella expansa*	*Paludicella articulata*
**lophophoral base**						
brain	1	1	1	1	1	1
circumoral nerve ring	1	1	1	1	1	1
outer nerve ring	1	?	?	?	0	1
trifid nerve	1	1	1	?	1	1
descending branch	?	?	1	?	?	?
annular branch	1	?	1	?	?	?
visceral branch	1	?	?	?	0	1
direct nerve origin sites	?	1	1	?	?	?
direct tentacle sheath nerve	1	1	1	?	1	1
**lophophore**						
radial nerves	1	?	?	1	1	1
abfrontal tentacle nerve	1	?	?	1	1	1
mediofrontal tentacle rootlets	1	?	?	?	1	1
mediofrontal tentacle nerve	1	?	?	1	1	1
laterofrontal tentacle nerve	1	?	?	1	1	1
intertentacular pits	1	?	1	1	?	1
intertentacular neurites	1	?	?	?	?	?
**visceral innervation**						
mediovisceral nerve	1	?	?	?	1	1
(pharyngeal) laterovisceral nerves	1	?	?	?	1	1
(pharyngeal) medio-laterovisceral nerve	1	?	?	?	?	1
pharyngeal plexus	1	?	?	?	1	1
visceral ganglion	?	?	?	?	?	?
**peripheral innervation**						
compound tentacle sheath nerve	?	?	1	?	?	?
parietal branching site	?	?	1	?	?	?
parietal muscle nerve	?	?	?	?	1	?
parietovestibular branch	0	0	0	0	0	0
sphincter branch	?	?	1	?	?	?
parietodiaphragmatic nerve	?	?	?	?	?	?
**remainder**						
interzooidal pores	?	?	?	?	?	?
		1	present			
		0	absent			
		?	missing data			

## Results

### Zooidal morphology

**Fig. 2:**
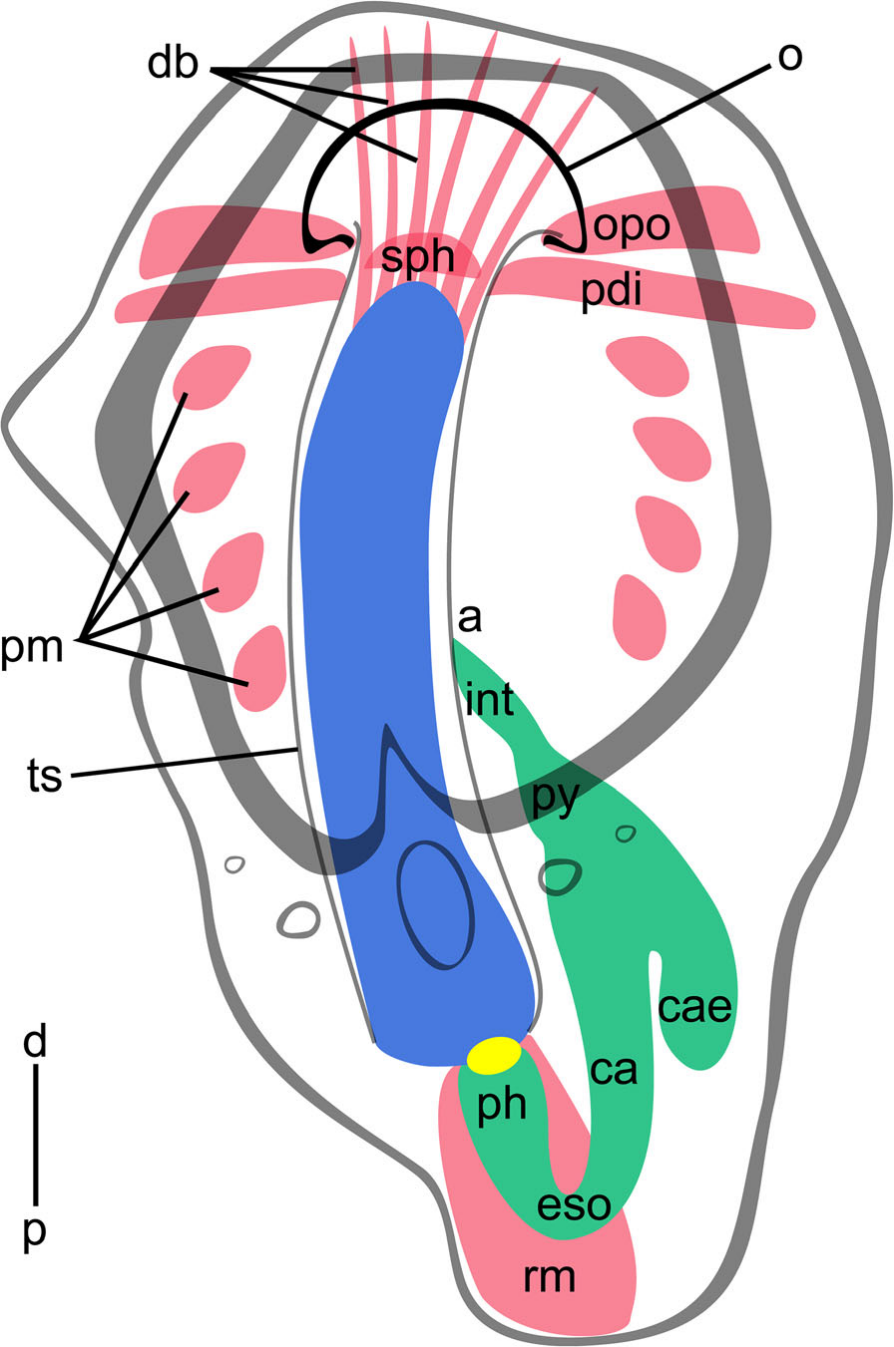
Schematic overview on the morphology of cheilostome bryozoans based on *Electra posidoniae* from the frontal side. The cystid (gray) is calcified. The polypide is composed of a lophophore (blue), digestive tract (green), musculature (pink) and the nervous system (cerebral ganglion in yellow). Abbreviations: a – anus, ca – cardia, cae – caecum, d – distal, db – duplicature bands, eso – esophagus, int – intestine, o – operculum, opo – operculum occlusor, p – proximal, pdi – parietodiaphragmatic muscle, ph – pharynx, pm – parietal muscle, py – pylorus, rm. – retractor muscle, sph – sphincter muscle, ts – tentacle sheath

**Fig. 3:**
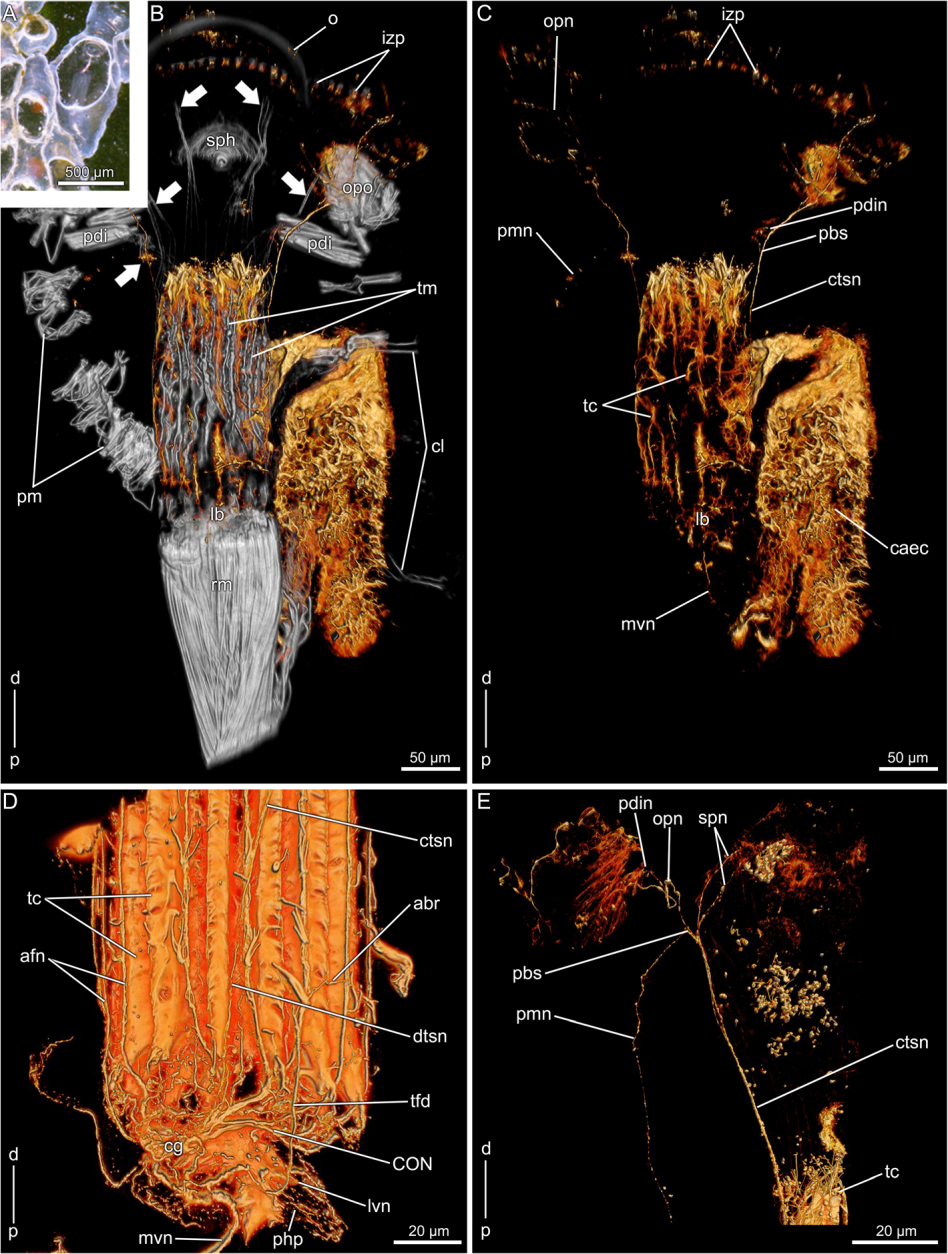
*Electra posidoniae*. Acetylated alpha-tubulin-lir in orange in all images. A Overview of few zooids. B Overview from the frontal side, showing alpha-tubulin-lir and f-actin (gray). Volume rendering. C Overview from the frontal side, showing alpha-tubulin-lir structures. Volume rendering. D Detail of the lophophoral base. Volume rendering. E Detail of the apertural area. Volume rendering. Abbreviations: abr – annular branch of the trifid neurite bundle, afn – abfrontal tentacle neurite bundle, caec – caecum cilia, cl – caecum ligament, cg – cerebral ganglion, CON – circumoral nerve ring, ctsn – compound tentacle sheath nerve, d – distal, izp – interzooidal pores, dtsn – direct tentacle sheath nerve, lb. – lophophoral base, lvn – laterovisceral neurite bundle, mvn – mediovisceral neurite bundle, o – operculum, opn – opercular nerve, opo – operculum occlusor, p – proximal, pbs – parietal branching site, pdi – parietodiaphragmatic muscle, pdin – parietodiaphragmatic nerve, php – pharyngeal nerve plexus, pm – parietal muscle, pmn – parietal muscle nerve, rm. – retractor muscle, sph – sphincter muscle, spn – sphincter nerve, tc – tentacle cilia, tfd – trifid neurite bundle, tm – tentacle muscle, white arrows indicate the position of duplicature bands

**Fig. 4:**
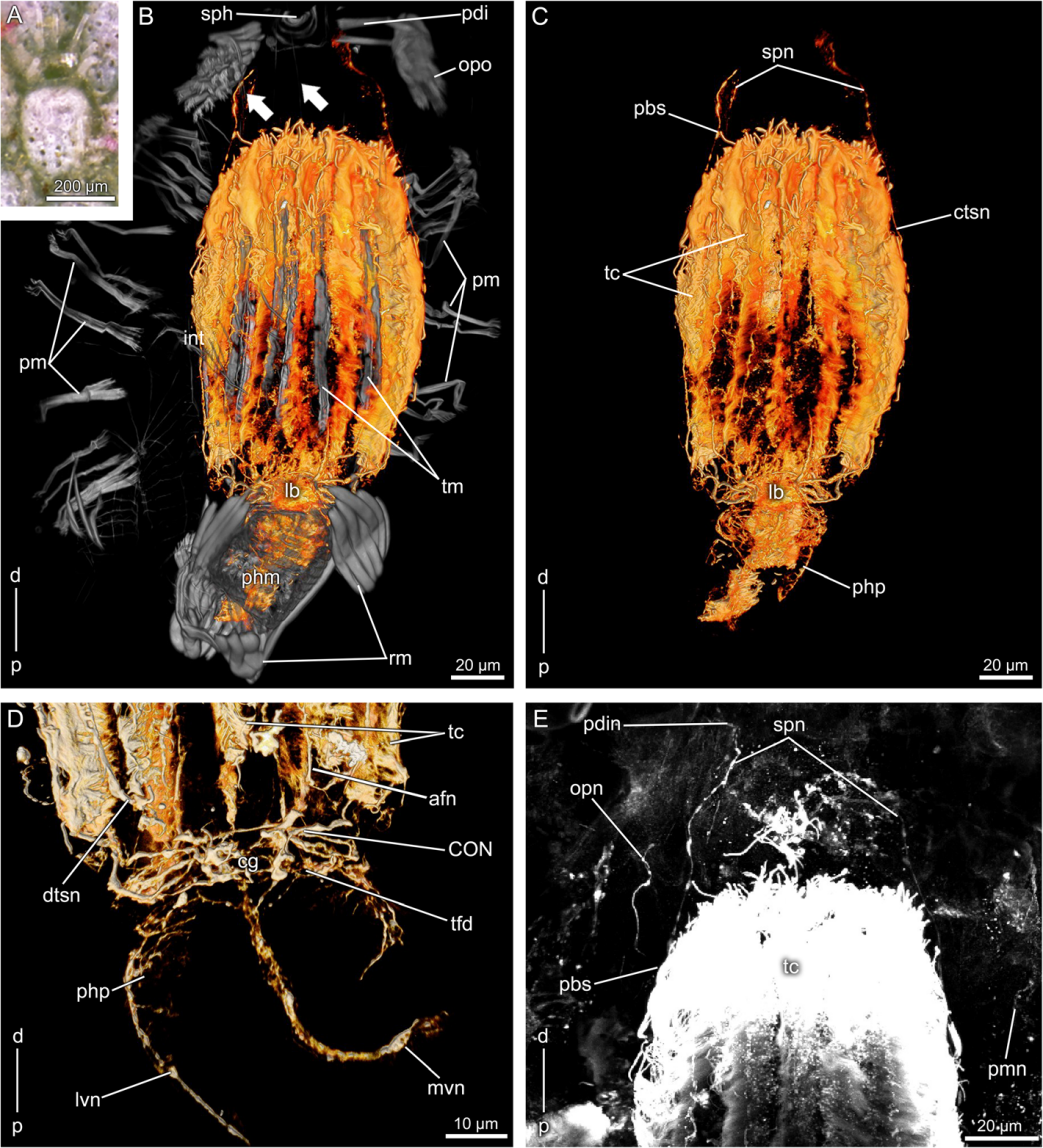
*Fenestrulina joannae*. A Overview of a zooid. B Overview from the frontal side, showing alpha-tubulin-lir in orange and f-actin in gray. Volume rendering. C Overview from the frontal side, showing alpha-tubulin-lir structures in orange. Volume rendering. D Detail of the lophophoral base. Alpha-tubulin-lir in orange. Volume rendering. E Detail of apertural area. Alpha-tubulin-lir in gray. Maximum intensity projection. Abbreviations: afn – abfrontal tentacle neurite bundle, cg – cerebral ganglion, CON – circumoral nerve ring, ctsn – compound tentacle sheath nerve, d – distal, dtsn – direct tentacle sheath nerve, int – intestine, lb. – lophophoral base, lvn – laterovisceral neurite bundle, mvn – mediovisceral neurite bundle, opn – opercular nerve, opo – operculum occlusor, p – proximal, pbs – parietal branching site, pdi – parietodiaphragmatic muscle, pdin – parietodiaphragmatic nerve, phm – pharyngeal musculature, php – pharyngeal nerve plexus, pm – parietal muscle, pmn – parietal muscle nerve, rm. – retractor muscle, sph – sphincter muscle, spn – sphincter nerve, tc – tentacle cilia, tfd – trifid neurite bundle, tm – tentacle muscle

**Fig. 5:**
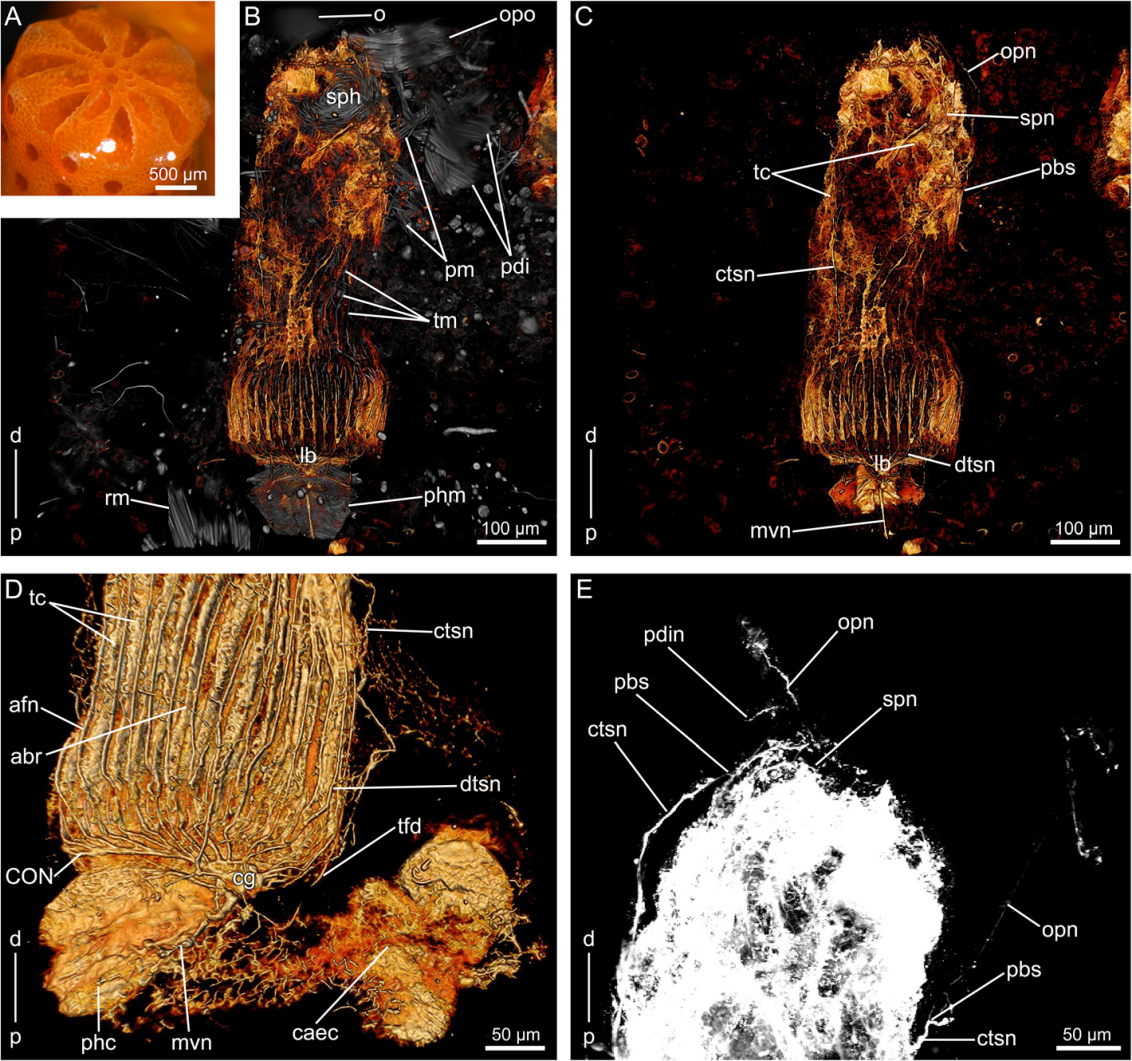
*Myriapora truncata*. A Overview of a few zooids. B Overview from the basal side, showing alpha-tubulin-lir in orange and f-actin in gray. Volume rendering. C Overview from the basal side, showing alpha-tubulin-lir structures in orange. Volume rendering. D Detail of the lophophoral base. Lateral view, alpha-tubulin-lir in orange. Volume rendering. E Detail of apertural area. Alpha-tubulin-lir in gray Maximum intensity projection. Abbreviations: abr – annular branch of trifid neurite bundle, afn – abfrontal tentacle neurite bundle, caec – caecum cilia, cg – cerebral ganglion, CON – circumoral nerve ring, ctsn – compound tentacle sheath nerve, d – distal, dtsn – direct tentacle sheath nerve, lb. – lophophoral base, mvn – mediovisceral neurite bundle, o – operculum, opn – opercular nerve, opo – operculum occlusor muscle, p – proximal, pbs – parietal branching site, pdi – parietodiaphragmatic muscle, pdin – parietodiaphragmatic nerve, phc – pharyngeal cilia, phm – pharyngeal musculature, pm – parietal muscle, pmn – parietal muscle nerve, rm. – retractor muscle, sph – sphincter muscle, spn – sphincter nerve, tc – tentacle cilia, tfd – trifid neurite bundle, tm – tentacle muscle

**Fig. 6:**
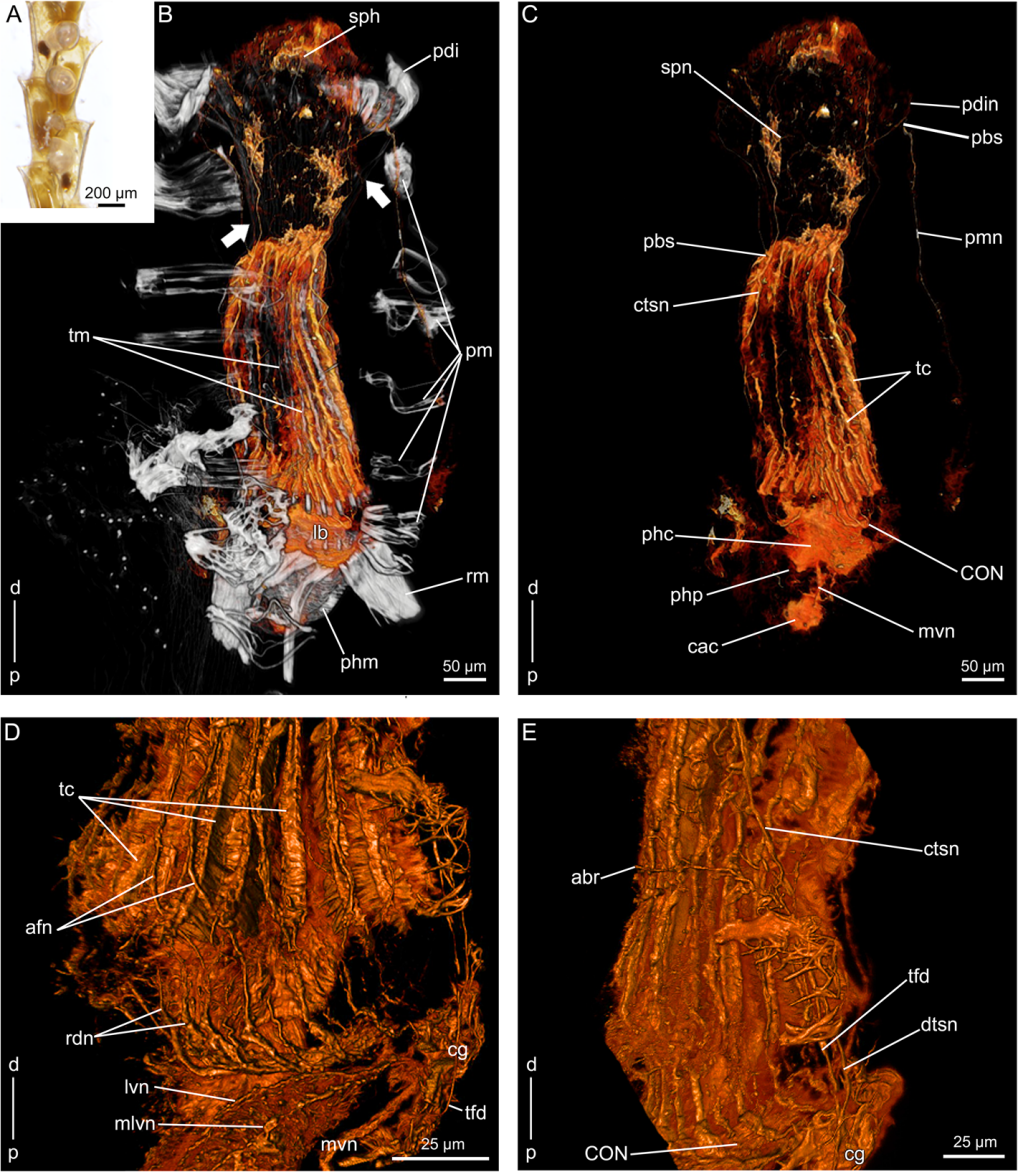
*Bugula neritina*. Acetylated alpha-tubulin-lir in orange in all images. A Overview of few zooids. B Overview from the frontal side, showing alpha-tubulin-lir and f-actin (gray). Volume rendering. C Overview from the frontal side, showing alpha-tubulin-lir structures. Volume rendering. D Detail of the lophophoral base in lateral view. Volume rendering. E Detail of apertural area in lateral view. Volume rendering. Abbreviations: abr – annular branch of trifid neurite bundle, afn – abfrontal tentacle neurite bundle, cac – cardia cilia, cg – cerebral ganglion, CON – circumoral nerve ring, ctsn – compound tentacle sheath nerve, d – distal, dtsn – direct tentacle sheath nerve, lb. – lophophoral base, lvn – laterovisceral neurite bundle, mlvn – mediolaterovisceral neurite bundle, mvn – mediovisceral neurite bundle, p – proximal, pbs – parietal branching site, pdi – parietodiaphragmatic muscle, pdin – parietodiaphragmatic nerve, phc – pharyngeal cilia, phm – pharyngeal musculature, php – pharyngeal nerve plexus, pm – parietal muscle, pmn – parietal muscle nerve, rdn – radial nerve, rm. – retractor muscle, sph – sphincter muscle, spn – sphincter nerve, tc – tentacle cilia, tfd – trifid neurite bundle, tm – tentacle muscle, white arrows indicate the position of duplicature bands

**Fig. 7:**
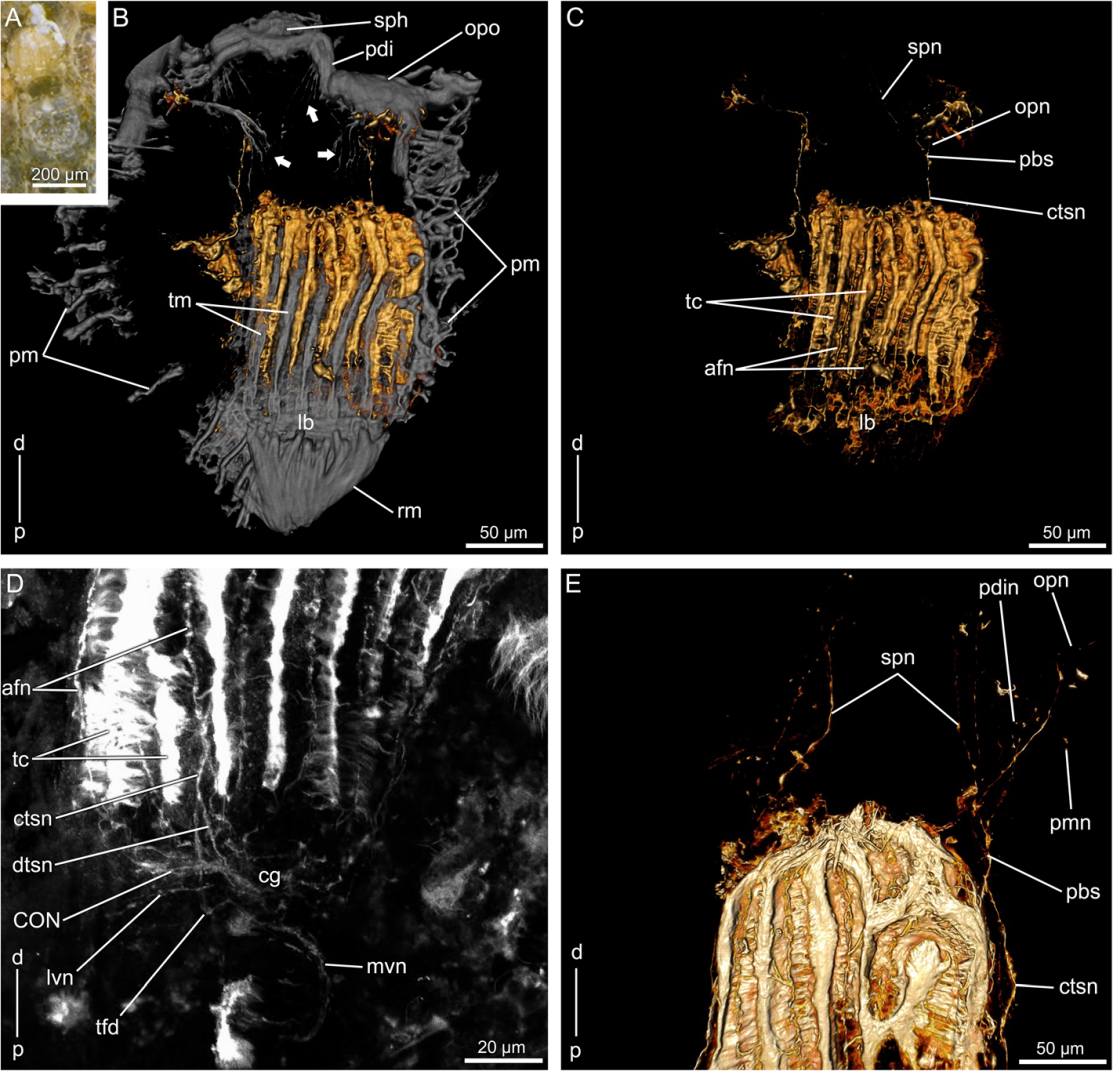
*Collarina balzaci*. A Overview of a zooid. B Overview from the frontal side, showing alpha-tubulin-lir in orange and f-actin in gray. C Overview from the frontal side, alpha-tubulin-lir in orange. D Detail of the lophophoral base, alpha-tubulin-lir in gray. Maximum intensity projection. E Detail of apertural area, alpha-tubulin-lir in orange. Volume rendering. Abbreviations: afn – abfrontal tentacle neurite bundle, cg – cerebral ganglion, CON – circumoral nerve ring, ctsn – compound tentacle sheath nerve, d – distal, dtsn – direct tentacle sheath nerve, lb. – lophophoral base, lvn – laterovisceral neurite bundle, mvn – mediovisceral neurite bundle, opn – opercular nerve, opo – operculum occlusor, p – proximal, pbs – parietal branching site, pdi – parietodiaphragmatic muscle, pdin – parietodiaphragmatic nerve, pm – parietal muscle, pmn – parietal muscle nerve, rm. – retractor muscle, sph – sphincter muscle, spn – sphincter nerve, tc – tentacle cilia, tfd – trifid neurite bundle, tm – tentacle muscle, white arrows indicate the position of duplicature bands

**Fig. 8:**
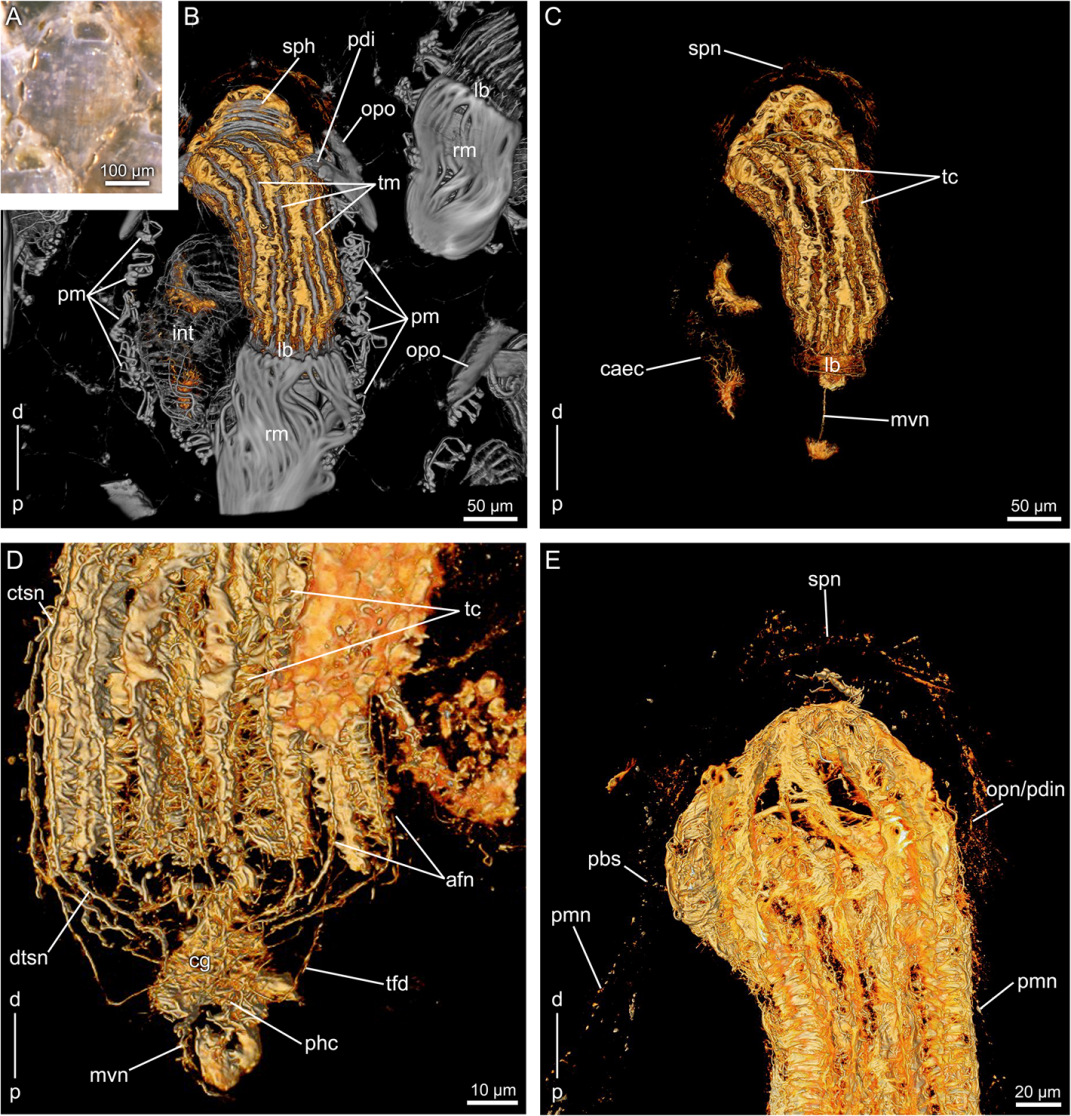
*Chorizopora brongniartii*. Acetylated alpha-tubulin-lir in orange in all images. A Overview of a zooid. F-actin in gray. B Overview from the frontal side, showing alpha-tubulin-lir and f-actin structures. Volume rendering. C Overview from the frontal side, showing alpha-tubulin-lir structures. Volume rendering. D Detail of the lophophoral base from the anal side. Volume rendering. E Detail of apertural area. Volume rendering. Abbreviations: afn – abfrontal tentacle neurite bundle, caec – caecum cilia, cg – cerebral ganglion, ctsn – compound tentacle sheath nerve, d – distal, dtsn – direct tentacle sheath nerve, int – intestine, lb. – lophophoral base, mvn – mediovisceral neurite bundle, opn – opercular nerve, opo – operculum occlusor, p – proximal, pbs – parietal branching site, pdi – parietodiaphragmatic muscle, pdin – parietodiaphragmatic nerve, phc – pharyngeal cilia, pm – parietal muscle, pmn – parietal muscle nerve, rm. – retractor muscle, sph – sphincter muscle, spn – sphincter nerve, tc – tentacle cilia, tfd – trifid neurite bundle, tm – tentacle muscle

In all analyzed species the operculum, a reinforced, cuticular rim for aperture/orifice closure, is located distally at the frontal side of each zooid (Fig. 2). In *Electra posidoniae,* it is autofluorescent (Fig. 3B). Prominent operculum occlusor muscles attach to the operculum on its lateral sides. The orifice continues into the vestibulum, a cavity lined by the vestibular wall, which terminates at the diaphragm marked by the diaphragmatic sphincter muscle (Figs. 2, 3B). The tentacle sheath extends from the diaphragm towards the lophophoral base and in case of retracted zooids enwraps the retracted lophophore. Duplicature bands supplied with thin longitudinal muscles connect the tentacle sheath with the lateral cystid wall (Figs. 2, 3B). Close to the cystid wall on both lateral sides of each zooid, a series of transversal parietal muscles is present. The tentacle sheath terminates at the lophophoral base, where prominent retractor muscles attach (Fig. 3B). From the lophophoral base, the mouth opening continues to the digestive tract, which commences with a pharynx with prominent ring muscles. An esophagus follows the pharynx until the cardia valve that separates the foregut from the midgut, which starts with a tubular cardia that enters the voluminous caecum. Two caecum ligaments project from its lateral side to the body wall in *E. posidoniae* (Fig. 3B). Apart from the caecum ligaments, the morphology of *E. posidoniae* is similar to conditions exhibited in *Fenestrulina joannae* (Fig. 4B) and *Myriapora truncata* (Fig. 5B). *Bugula neritina* lacks an operculum occlusor (Fig. 6B) and the visceral plexus is absent in *Collarina balzaci* (Fig. 7B). In contrast, *Chorizopora brongniartii* has a prominent muscular plexus around the digestive tract (Fig. 8B).

### Neuroanatomy of investigated species

#### Cerebral ganglion and directly emanating neurite bundles

**Fig. 9:**
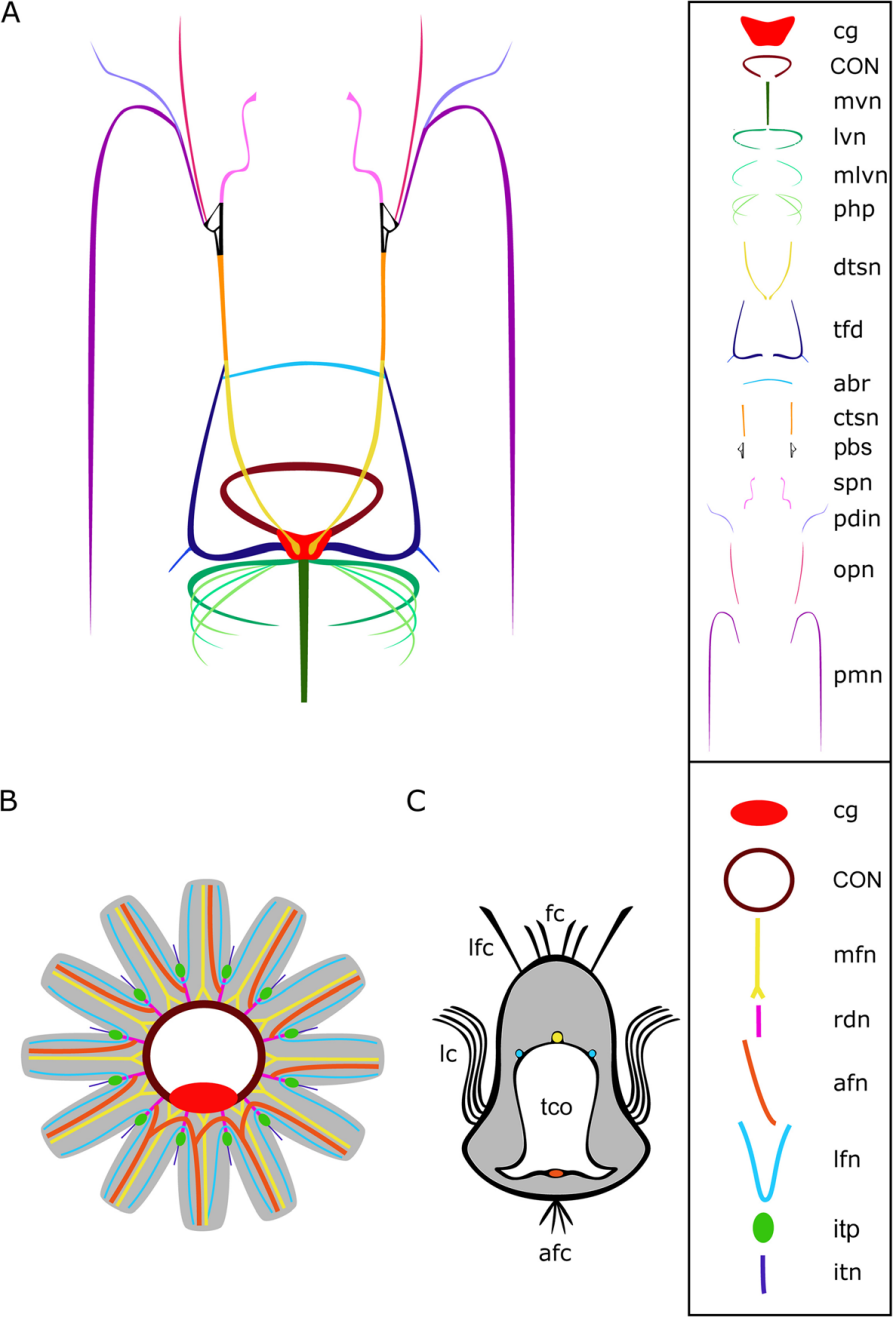
Ground pattern of the autozooidal innervation in cheilostomes. A Lateral view of the autozooidal innervation pattern without lophophore. B View from the distal side showing the innervation pattern of the lophophore C Cross section of a tentacle. Abbreviations: abr – annular branch of trifid nerve, afc – abfrontal cilia, afn – abfrontal tentacle neurite bundle, cg – cerebral ganglion, CON – circumoral nerve ring, ctsn – compound tentacle sheath nerve, dtsn – direct tentacle sheath nerve, fc – frontal cilia, itn – intertentacular neurite, itp – intertentacular pit, lc – lateral cilia, lfc – laterofrontal cilia, lfn – laterofrontal tentacle neurite bundle, lvn – laterovisceral neurite bundle, mfn – mediofrontal tentacle neurite bundle, mlvn – mediolaterovisceral neurite bundle, mvn – mediovisceral neurite bundle, pbs – parietal branching site, pdin – parietodiaphragmatic nerve, php – pharyngeal nerve plexus, pmn – parietal muscle nerve, opn – opercular nerve, rdn – radial nerve, spn – sphincter nerve, tco – tentacle coelom, tfd – trifid neurite bundle

**Fig. 10:**
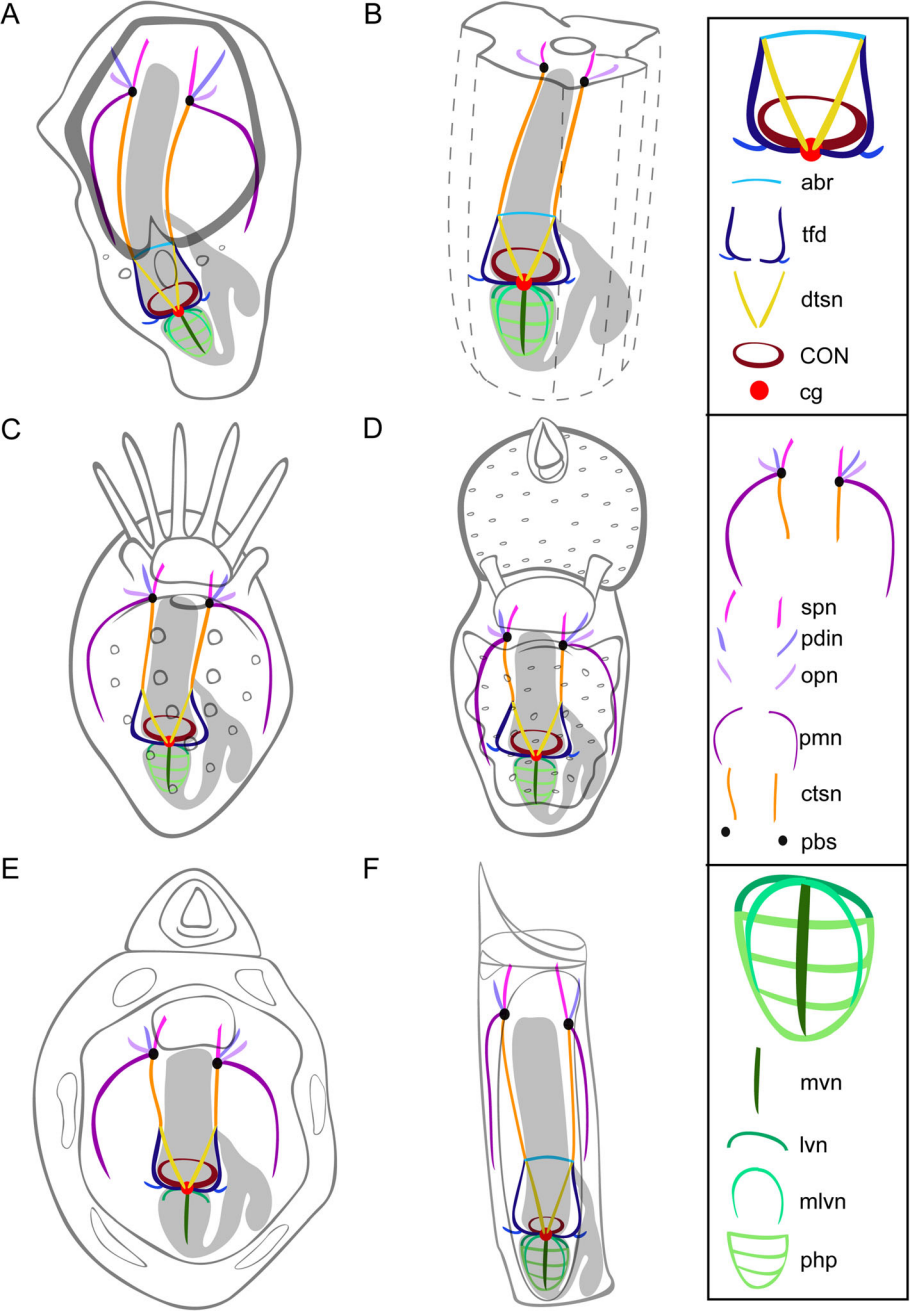
Innervation schemes shown from the frontal side based on data from this study. A *Electra posidoniae*. B *Myriapora truncata*. C *Fenestrulina joannae*. D *Collarina balzaci*. E *Chorizopora brongniartii*. F *Bugula neritina*. Abbreviations: abr – annular branch of trifid nerve, cg – cerebral ganglion, CON – circumoral nerve ring, ctsn – compound tentacle sheath nerve, dtsn – direct tentacle sheath nerve, lvn – laterovisceral neurite bundle, mlvn – mediolaterovisceral neurite bundle, mvn – mediovisceral neurite bundle, opn – opercular nerve, pbs – parietal branching site, pdin – parietodiaphragmatic nerve, php – pharyngeal nerve plexus, pmn – parietal muscle nerve, spn – sphincter nerve, tfd – trifid neurite bundle

The cerebral ganglion is situated at the lophophoral base (Figs. 3D, 4D, 5D, 6D, 7D, 8D, 9A, 10). From the cerebral ganglion, neurite bundles embrace the mouth opening as circumoral nerve ring (CON, Figs. 3D, 4D, 5D, 6E, 7D, 9A, B, 10). Two thick direct tentacle sheath neurite bundles emanate from the cerebral ganglion distally into the inverted tentacle sheath (Figs. 3D, 4D, 5D, 6E, 7D, 8D, 9A). A pair of trifid neurite bundles emanates from the lateral sides of the cerebral ganglion (Figs. 3D, 4D, 5D, 6E, 7D, 8D, 9A, 10). Shortly after, each bundle bifurcates with one branch projecting towards the vestibulum (Figs. 3D, 4D, 5D, 7D, 8D, 10), and fuses close to the lophophoral base with the direct tentacle sheath neurite bundles to form the compound tentacle sheath neurite bundle (Figs. 7D, 9A). Shortly before their fusion, neurite bundles from the trifid tentacle sheath branch emanate laterally to an annular branch forming an open circle around the base of the lophophore (Figs. 3D, 5D, 6E, 9A). The annular branch of the trifid neurite bundle is absent in *Fenestrulina joannae*, *Collarina balzaci* and *Chorizopora brongniartii* (Table. 1). The second branch of the trifid neurite bundle, the descending branch, bends proximally at the lateral branching point (Figs. 3D, 5D, 9A, 10). These so-called descending branches of the trifid neurite bundles are absent in *F. joannae* (Table. 1).

#### Visceral innervation

**Fig. 11:**
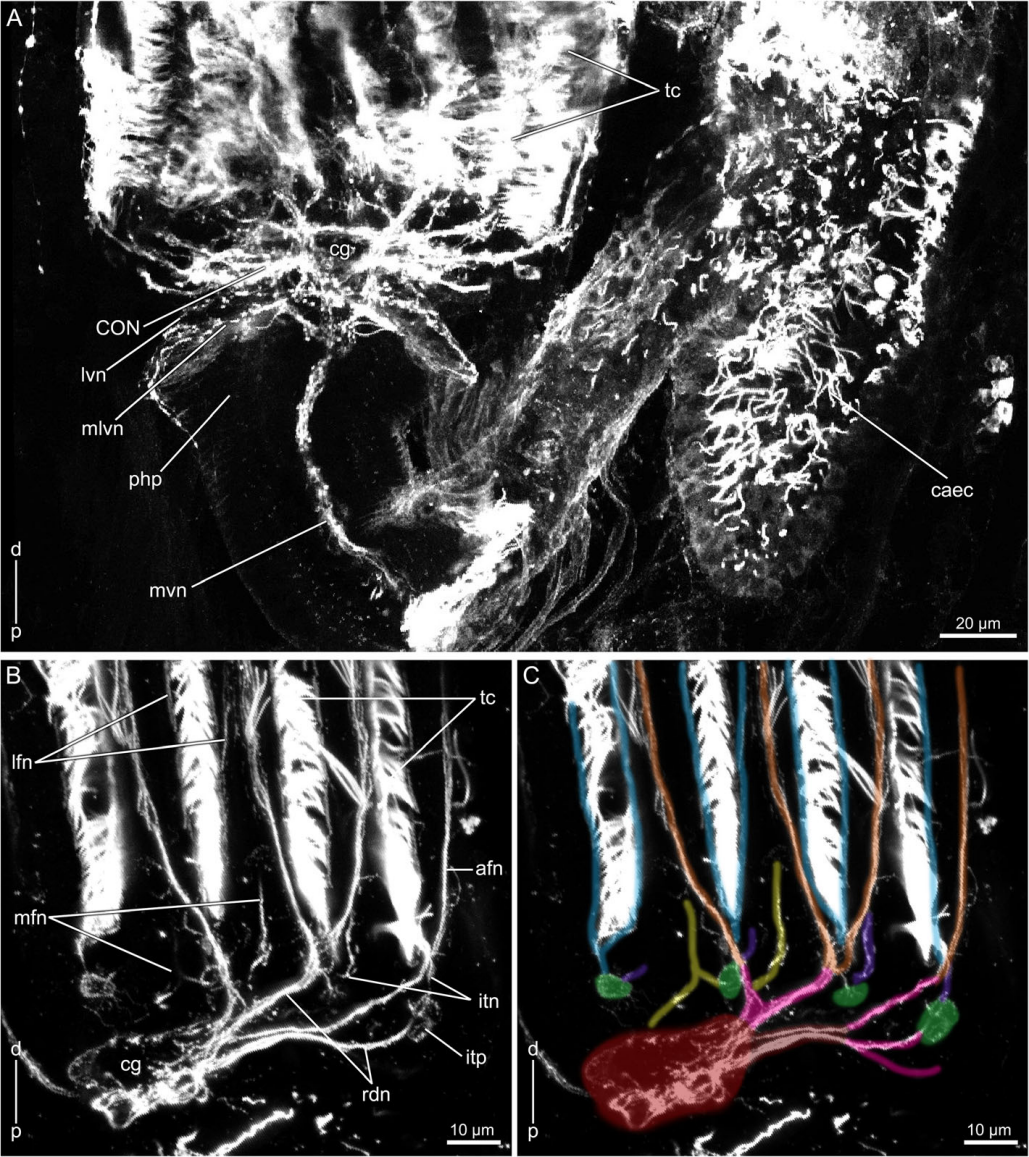
Acetylated alpha-tubulin-lir (gray) in the lophophore of *Electra posidoniae*. A Detail of the lophophoral base and of the digestive tract. View from anal side of lophophoral base. B Detail of tentacular innervation at the lophophoral base. C Same as in B but labeled with a color code. Abbreviations and color code: afn – abfrontal tentacle neurite bundle (orange), caec – caecal cilia, cg – cerebral ganglion (red), CON – circumoral nerve ring (brown), d – distal, itn – intertentacular neurite (purple), itp – intertentacular pit (green), lfn – laterofrontal tentacle neurite bundle (blue), lvn – laterovisceral neurite bundle, mfn – mediofrontal tentacle neurite bundle (yellow), mlvn – mediolaterovisceral neurite bundle, mvn – mediovisceral neurite bundle, p – proximal, php – pharyngeal nerve plexus, rdn – radial nerve (pink), tc – tentacle cilia

**Fig. 12:**
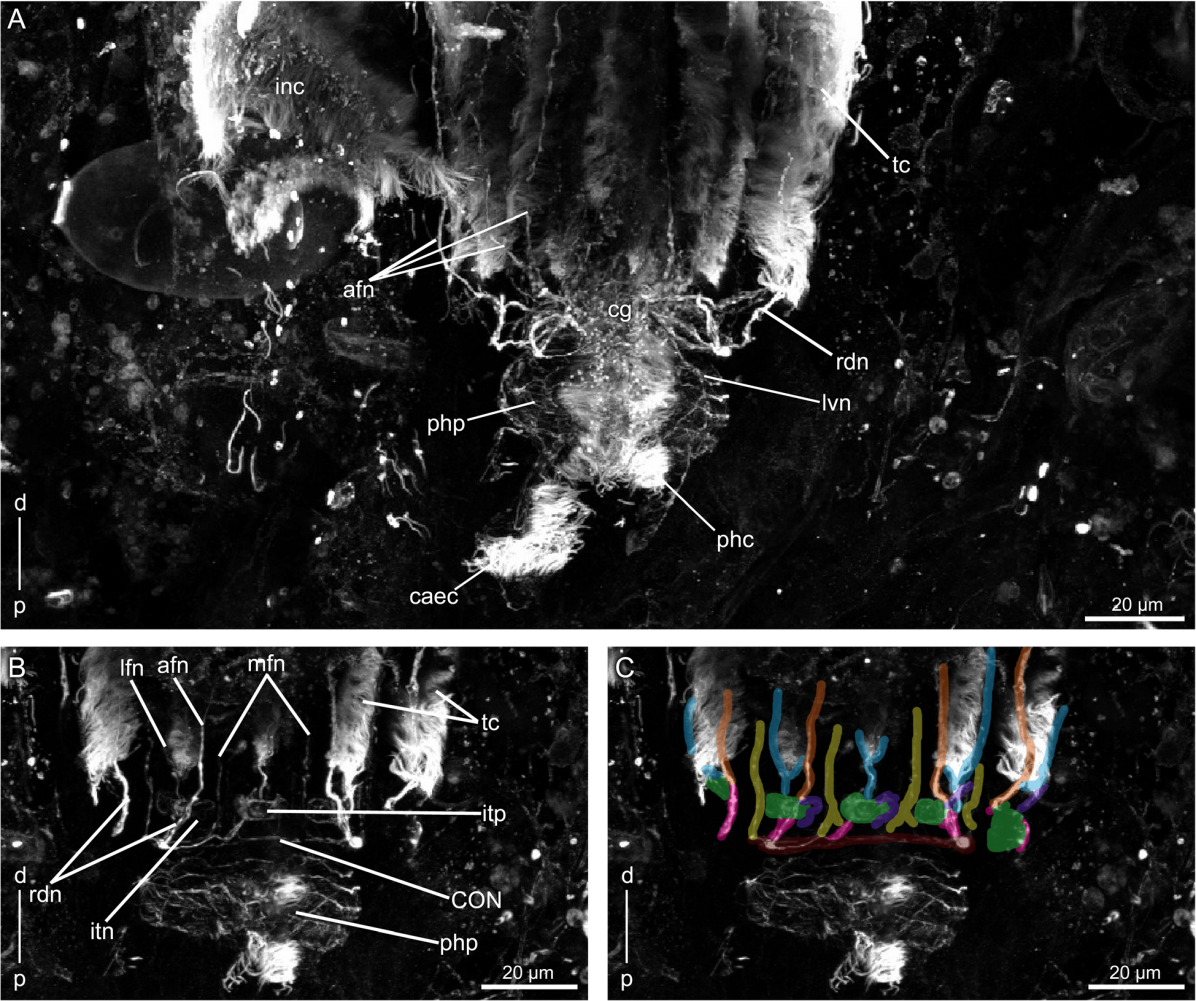
Acetylated alpha-tubulin-lir (gray) in the lophophore of *Fenestrulina joannae*. A Detail of the lophophoral base and the visceral tract. B Detail of tentacular innervation at the lophophoral base. C Same as in B but labeled with a color code. Abbreviations and color code: afn – abfrontal tentacle neurite bundle (orange), caec – cardia cilia, cg – cerebral ganglion (red), CON – circumoral nerve ring (brown), d - distal, itn – intertentacular neurite (purple), inc – intestinal cilia, itp – intertentacular pit (green), lfn – laterofrontal tentacle neurite bundle (blue), lvn – laterovisceral neurite bundle, mfn – mediofrontal tentacle neurite bundle (yellow), p – proximal, phc – pharyngeal cilia, php – pharyngeal nerve plexus, rdn – radial nerve (pink), tc – tentacle cilia

**Fig. 13:**
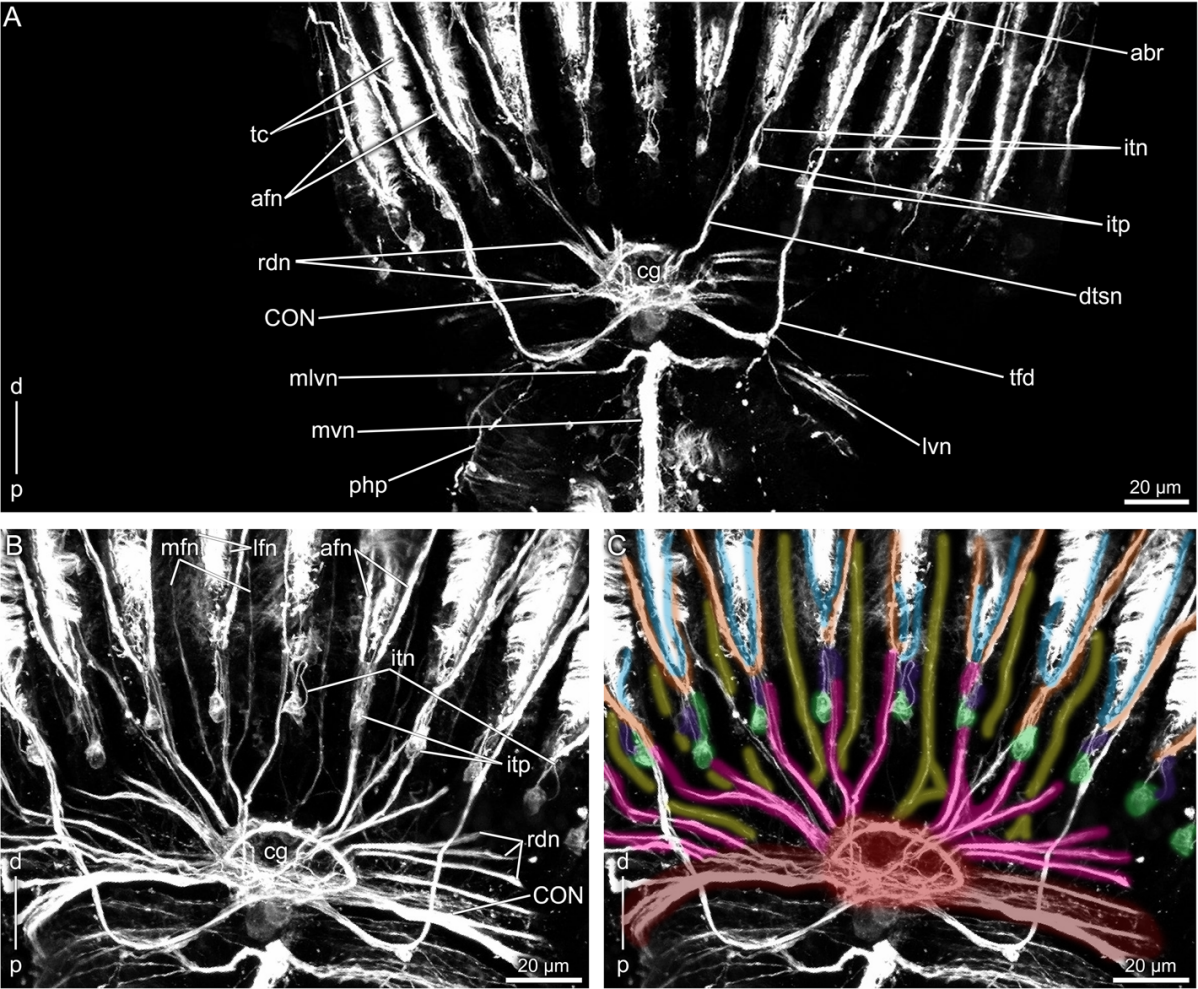
Acetylated alpha-tubulin-lir (gray) in the lophophore of *Myriapora truncata*. A Detail of the lophophoral base. B Detail of tentacular innervation at the lophophoral base. C Same as in B but labeled with a color code. Abbreviations and color code: abr – annular branch of trifid nerve, afn – abfrontal tentacle neurite bundle (orange), cg – cerebral ganglion (red), CON – circumoral nerve ring (brown), d – distal, dtsn – direct tentacle sheath nerve, itn – intertentacular neurite (purple), itp – intertentacular pit (green), lfn – laterofrontal tentacle neurite bundle (blue), lvn – laterovisceral nerve, mfn – mediofrontal tentacle nerve (yellow), mlvn – mediolaterovisceral neurite bundle, mvn – mediovisceral neurite bundle, p – proximal, php – pharyngeal nerve plexus, rdn – radial nerve (pink), tc – tentacle cilia, tfd – trifid neurite bundle

**Fig. 14:**
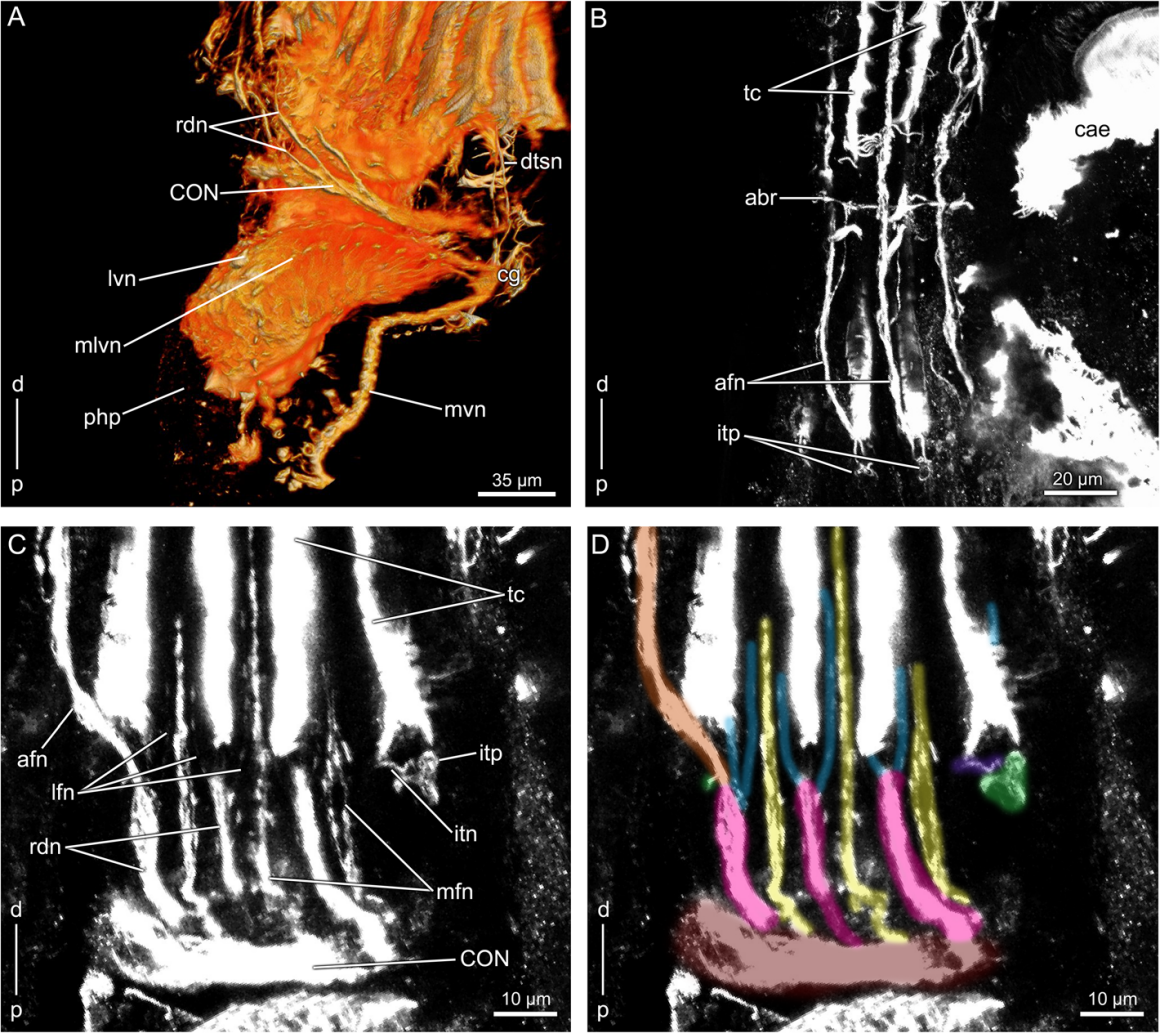
Acetylated alpha-tubulin-lir in the lophophore of *Bugula neritina*. A Detail of the lophophoral base. Acetylated alpha-tubulin-lir in orange. B Detail of the annular branch of trifid nerve. Acetylated alpha-tubulin-lir in gray. C Detail of tentacular innervation at the lophophoral base. Acetylated alpha-tubulin-lir in gray D Same as in C but labeled with a color code. Abbreviations and color code: abr – annular branch of trifid nerve, afn – abfrontal tentacle neurite bundle (orange), cae – caecum, cg – cerebral ganglion (red), CON – circumoral nerve ring (brown), d – distal, dtsn – direct tentacle sheath nerve, itn – intertentacular neurite (purple), itp – intertentacular pit (green), lfn – laterofrontal tentacle neurite bundle (blue), lvn – laterovisceral neurite bundle, mfn – mediofrontal tentacle neurite bundle (yellow), mlvn – mediolaterovisceral neurite bundle, mvn – mediovisceral neurite bundle, p – proximal, php – pharyngeal nerve plexus, rdn – radial nerve (pink), tc – tentacle cilia

**Fig. 15:**
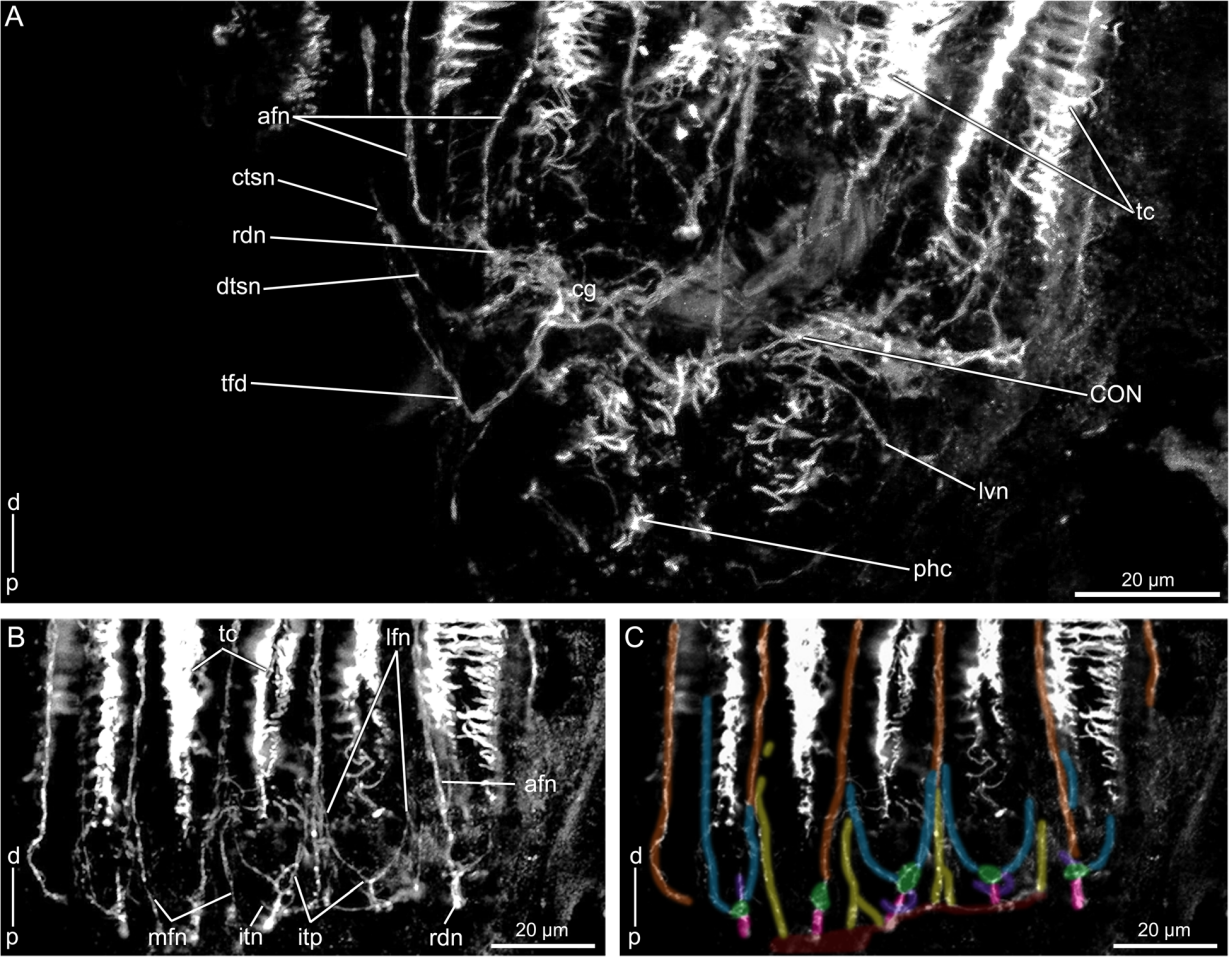
Acetylated alpha-tubulin-lir (gray) in the lophophore of *Collarina balzaci*. A Detail of the lophophoral base. B Detail of tentacular innervation at the lophophoral base. C Same as in B but labeled with a color code. Abbreviations and color code: afn – abfrontal tentacle neurite bundle (orange), cg – cerebral ganglion (red), CON – circumoral nerve ring (brown), ctsn – compound tentacle sheath nerve, d – distal, dtsn – direct tentacle sheath nerve, itn – intertentacular neurite (purple), itp – intertentacular pit (green), lfn – laterofrontal tentacle neurite bundle (blue), lvn – laterovisceral neurite bundle, mfn – mediofrontal tentacle neurite bundle (yellow), p – proximal, phc – pharyngeal cilia, php – pharyngeal nerve plexus, rdn – radial nerve (pink), tc – tentacle cilia, tfd – trifid neurite bundle

**Fig. 16:**
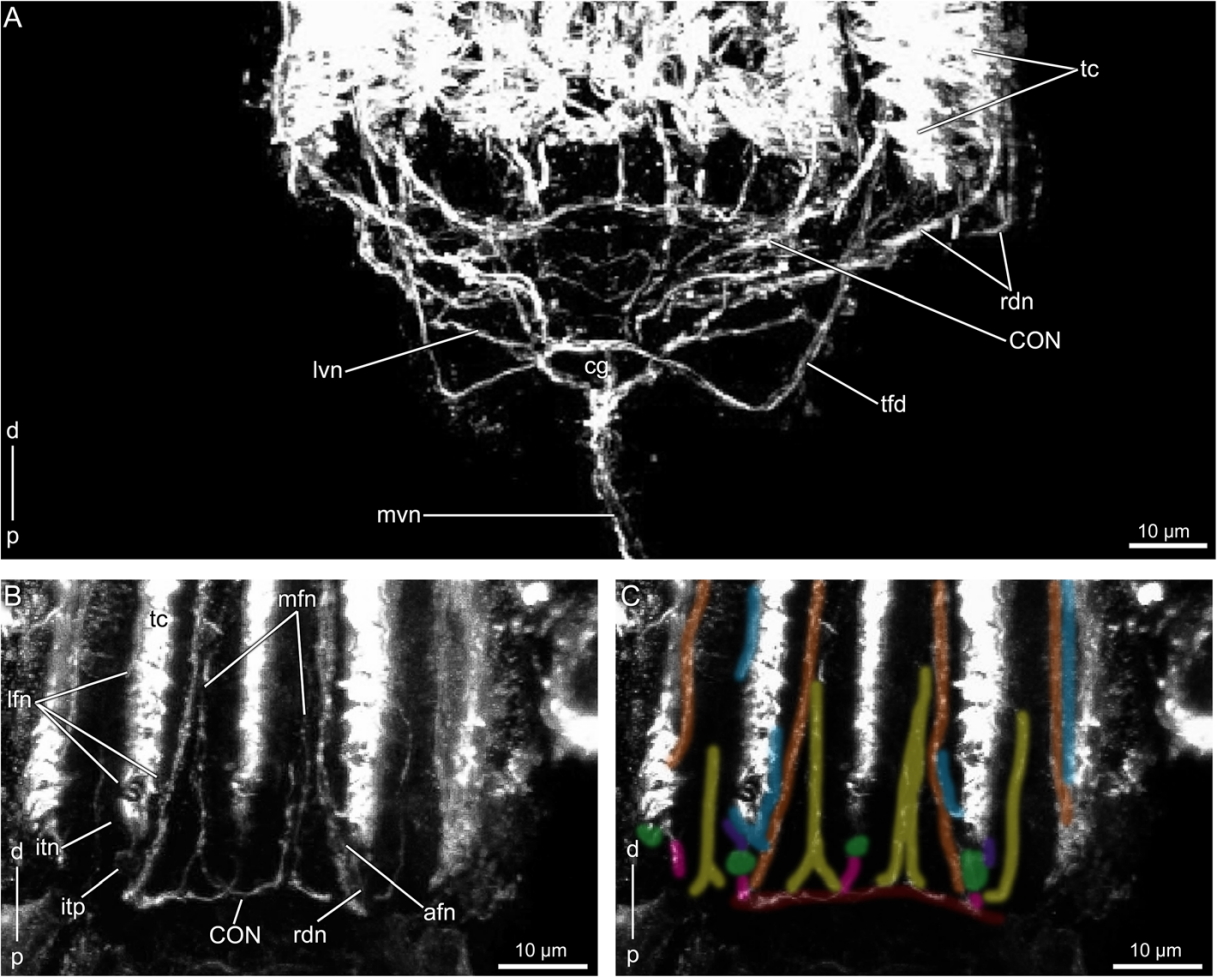
Acetylated alpha-tubulin-lir (gray) in the lophophore of *Chorizopora brongniartii*. A Detail of the lophophoral base. B Detail of tentacular innervation at the lophophoral base. C Same as in B but labeled with a color code. Abbreviations and color code: afn – abfrontal tentacle neurite bundle (orange), cg – cerebral ganglion (red), CON – circumoral nerve ring (brown), d – distal, itn – intertentacular neurite (purple), itp – intertentacular pit (green), lfn – laterofrontal tentacle neurite bundle bundle (blue), lvn – laterovisceral neurite bundle, mfn – mediofrontal tentacle nerve (yellow), mvn – mediovisceral neurite bundle, p – proximal, rdn – radial nerve (pink), tc – tentacle cilia, tfd – trifid neurite bundle

The cerebral ganglion gives rise to a mediovisceral neurite bundle in proximal direction, which extends along the anal side of the pharynx (Figs. 3D, 4D, 5D, 6D, 7D, 8D, 9A, 10). Two laterovisceral- and two mediolaterovisceral neurite bundles additionally innervate the muscular pharynx from the cerebral ganglion (Figs. 10, 11A, 12A, 13A, 14A, 15A, 16A). Mediolaterovisceral neurite bundles are absent in *Fenestrulina joannae*, *Collarina balzaci*, and *Chorizopora brongniartii*. All visceral neurite bundles terminate at the proximal end of the foregut (Figs. 5D, 7D, 8C, 10, 11A, 12A, 14A). A pharyngeal nerve plexus surrounds the pharynx and interconnects the visceral neurite bundles (Figs. 3D, 10, 12A, 13A, 14A, A). The pharyngeal nerve plexus is absent in *C. brongniartii* (Table. 1).

#### Peripheral innervation

The compound tentacle sheath neurite bundles follow the basal duplicature bands and terminate close to the diaphragm on each lateral side at a parietal branching point (Figs. 3E, 4E, 5E, 6C, 7E, 8E, 9A, 10). Each bundle splits from the latter into an operculum occlusor neurite bundle, a parietodiaphragmatic neurite bundle, a sphincter neurite bundle, and a parietal muscle neurite bundle (Figs. 3E, 4E, 5E, 6C, 7E, 8E, 9A, 10). The parietal muscles are innervated by one main parietal muscle neurite bundle, from which smaller neurite bundles project to the respective muscles (Figs. 3E, 6C, 8E, 9A, 10). Besides the parietal nerve, *Electra posidoniae* shows acetylated alpha-tubulin-like-immunoreactivity (lir) in interzooidal pores (Fig. 3C), which is lacking in all other examined species (Table. 1).

The parietal muscle neurite bundles and parietodiaphragmatic neurite bundles were not detected in *Myriapora truncata* (Table. 1). The opercular neurite bundle is absent in *Bugula neritina* (Table. 1). Alpha-tubulin-lir is present in interzooidal pores of *Electra posidoniae* (Fig. 3C), but absent in the interzooidal pores of all remaining examined species (Table. 1).

#### Tentacular innervation

**Fig. 17:**
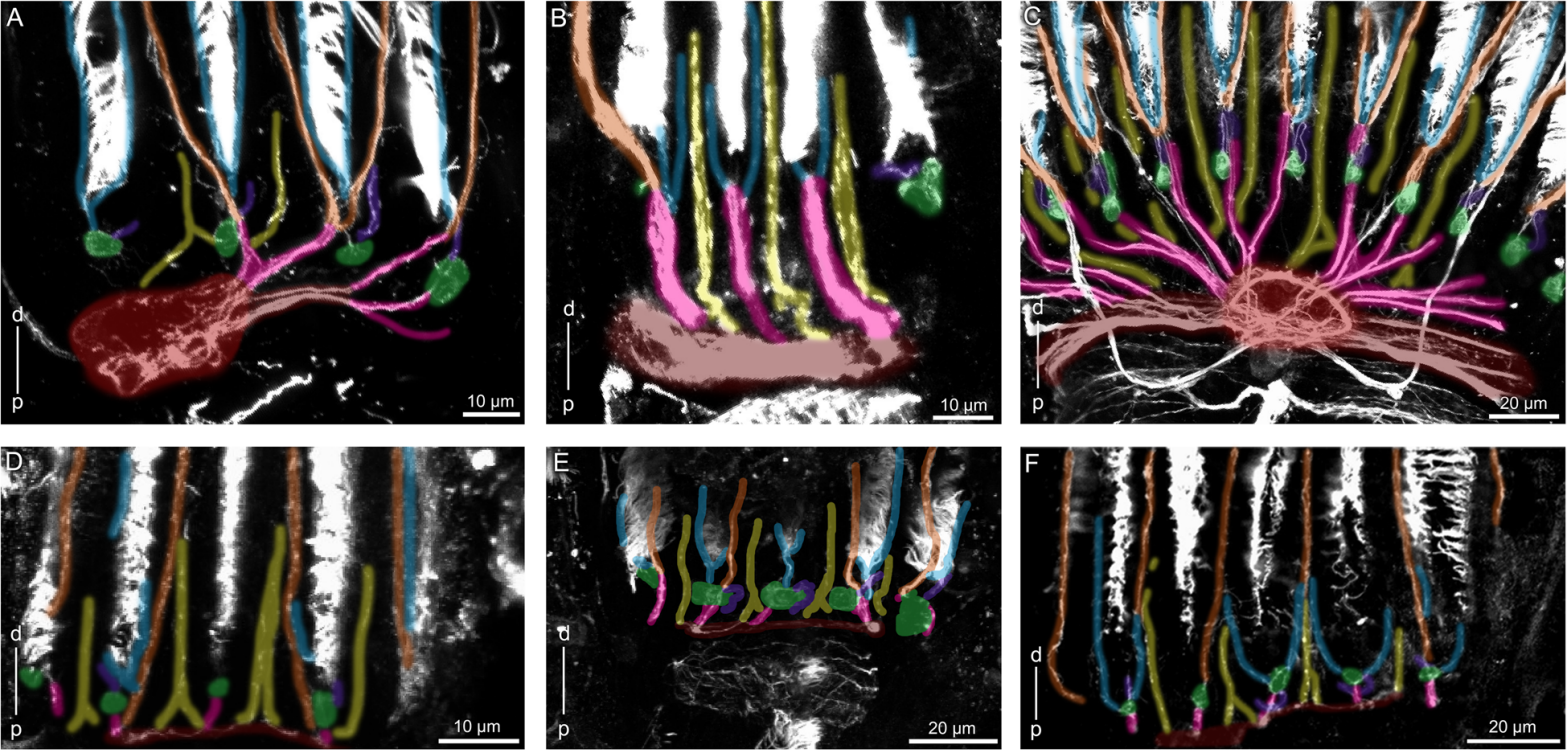
Comparison of the acetylated alpha-tubulin-lir (gray) in the lophophoral base of six cheilostome species. A & C are from the anal side showing the cerebral ganglion, B slightly laterally of the latter two showing a thick circum-oral nerve ring, and D-F are from the oral side where the nerve ring is only thin. A *Electra posidoniae*. B *Bugula neritina*. C *Myriapora truncata*. D *Chorizopora brongniartii*. E *Fenestrulina joannae*. F *Collarina balzaci*. Abbreviations and color code: abfrontal tentacle neurite bundle (orange), cerebral ganglion (red), circumoral nerve ring (brown), d – distal, intertentacular neurite (purple), intertentacular pit (green), laterofrontal tentacle neurite bundle (blue), mediofrontal tentacle neurite bundle (yellow), p – proximal, radial nerve (pink)

The cerebral ganglion and the circumoral nerve ring innervate the lophophore. Tentacle neurite bundles emerge directly from the cerebral ganglion on the anal side and from the nerve ring in the remaining parts of the lophophore (Figs. 9B, 17). Four neurite bundles innervate each tentacle (Figs. 9B, 11B,C, 12B,C, 13B,C, 14B,C, 15B,C, 16B,C). Mediofrontal tentacle neurite bundles emanate from the circumoral nerve ring directly into the center of each tentacle (Figs. 9B, 11B,C, 12B,C, 13B,C, 14B,C, 15B,C, 16B,C). Short ‘neurite bundles’ also project intertentacularly from the cerebral ganglion or circumoral nerve ring into distal direction, which are termed as ‘radial nerves’ (Figs. 9B, 11B,C, 12B,C, 13B,C, 14B,C, 15B,C, 16B,C). Few smaller neurites extend from the radial nerves into the intertentacular pits situated between the bases of adjacent tentacles (Figs. 9B, 11B,C, 12B,C, 13B,C, 14B,C, 15B,C, 16B,C). Abfrontal tentacle neurite bundles emanate from each radial nerve into each tentacle (Figs. 9B, 11B,C, 12B,C, 13B,C, 14B,C, 15B,C, 16B,C). The roots of these neurite bundles are paired in the area of the cerebral ganglion and thus symmetrical further distal merging in the median plane of each tentacle (Figs. 9B, 11B,C, 13B,C), whereas they are asymmetrical and single in the remaining lophophore (Figs. 9B, 12B,C, 14B,C, 15B,C, 16B,C). A pair of latero-frontal neurite bundles also emanates from the radial nerves into each tentacle (Figs. 9B, 11B,C, 12B,C, 13B,C, 14B,C, 15B,C, 16B,C).

## Discussion

### Cerebral ganglion and lophophoral base

The cerebral ganglion is situated on the anal side of the polypide at the base of the lophophore and varies in size from about 15 to 30 μm, which is similar to other bryozoans [see 33, 35]. The main neurite bundles originate from clusters of somata in different parts of the cerebral ganglion. The most prominent neurite bundle emanating from the cerebral ganglion is the circumoral nerve ring (CON), which occurs in all bryozoan taxa [4, 33, 35] and gives rise to the tentacle neurite bundles [4, 5].

The cerebral ganglion of ctenostome gymnolaemates is similar to cheilostomes, but sometimes can exhibit a cavity or lumen. The latter is present in all bryozoans during development, but in gymnolaemates only persists in few ctenostomes analyzed so far [4, 26]. Recently, such a cavity was also found in a cyclostome bryozoan [24]. The structure of the cyclostome cerebral ganglion is similar to that of gymnolaemates except for the presence of two associated lateral lobes [23, 24, 36]. The cerebral ganglia of the myolaemate sister-group, the Phylactolaemata, always contain a lumen. This indicates that the development of this lumen belongs to the bryozoan ground pattern and that it subsequently was lost in many myolaemate lineages including all cheilostomes [this study].

### Tentacle sheath innervation, the trifid and outer ring nerve

The tentacle sheath of myolaemates generally possesses two neurite bundles on the anal side [4, 23]. In addition to the direct tentacle sheath nerve, most gymnolaemates have an additional branch of the trifid neurite bundle that fuses with the direct one to form the compound tentacle sheath nerve [see 35, this study for cheilostomes]. This corresponds to early findings in the ctenostome *Flustrellidra hispida* [37, 38] and the cheilostomes *Membranipora membranacea* [37] and *Electra pilosa* [39].

An additional annular branch extends from the trifid neurite bundle to surround the lophophore proximally in form of an open ring, which has been described in other gymnolaemates [26–28, 38–40]. The annular branch is absent in the cheilostome *Chartella papyracea* [41] and in *Chorizopora brongniartii*, *Collarina balzaci* and *Fenestrulina joannae,* analyzed in this study (Table. 1), which might be an indication to miniaturization as they also possess distinctly smaller polypides. A distinct trifid neurite bundle is missing in cyclostomes and phylactolaemates, which might represent an apomorphy of gymnolaemates. However, an either complete or incomplete outer nerve ring was identified in ctenostomes [23, 27] and cyclostomes [23, 24], which originates from the lateral parts of the cerebral ganglion in an identical position as the gymnolaemate trifid neurite bundle and projects in parallel to the CON [23, 24, 27]. Some ctenostomes [compare 28] and all cheilostomes lack such a ring nerve. However, its distribution among bryozoan taxa indicates that it is present in the ground pattern of myolaemates and was subsequently altered to the trifid neurite bundle in gymnolaemates [4]. Phylactolaemates lack an outer ring nerve.

### Visceral innervation

The visceral innervation of gymnolaemates comprises a pharyngeal plexus and three different longitudinal visceral neurite bundles (VCB) that are either connected with the bundles of the plexus or with the trifid neurite bundle [see 33, 35]. Based on their position, these VCBs are termed medio-visceral (m-VCB), medio-lateral (ml-VCB), and latero-visceral (l-VCB) neurite bundles. All of these elements were found in cheilostome bryozoans [35, 42] and can be confirmed in different species showing some variation by the present study. A pharyngeal plexus is missing only in *Chorizopora brongniartii*, and the amount of VCBs varies from five in *Bugula neritina*, *Electra posidoniae* and *Myriapora truncata* to three in *Chorizopora brongniartii*, *Collarina balzaci* and *Fenestrulina joannae*.

The situation in ctenostome gymnolaemates is generally similar to cheilostomes [26–29]. All share the peripharyngeal plexus and the main median and the two l-VCB. The latter are generally associated with the pharyngeal plexus. In different species, different neurite bundles have been assigned as ml-VCB – the criterion being their position between the m-VCB and the l-VCBs. In the here analyzed cheilostomes, the m- and ml-VCBs are associated with the pharyngeal plexus, whereas ctenostomes show a higher variability concerning their number and association, either with the pharyngeal plexus or the trifid neurite bundle. M- and ml-VCBs can be lacking in e.g. *Hypophorella expansa* [28], range from two trifid and two pharyngeal ones in e.g. *Amathia gracilis* [27] or from three to five mostly trifid associated bundles in e.g. *Paludicella articulata* [26].

The two investigated cyclostome bryozoans, *Crisia eburnea* and *Cinctipora elegans*, possess a pharyngeal plexus, the m-VCB and two lateral ones, similar to gymnolaemates [23–25]. The visceral innervation of phylactolaemates extends over the pharynx to the cardia as a plexus with two prominent neurite bundles [20, 21]. In summary, a pharyngeal plexus is present in all bryozoans, most conspicuous in phylactolaemates and less prominent in myolaemates. The latter show more condensed neurite bundles in form of a series of longitudinal visceral bundles of various numbers on the anal to lateral side of the pharynx.

### Peripheral innervation

In cheilostomes, the compound tentacle sheath neurite bundles branche close to the aperture to innervate aperture associated- and parietal musculature. *Electra pilosa* possesses several branching areas to supply the respective muscles [43], whereas only a single branching area was identified in the cheilostomes of the current study. The peripheral innervation pattern of cheilostomes is similar to that of the ctenostome *Paludicella articulata* [26], whereas the ctenostome *Hypophorella expansa* only has the direct tentacle sheath neurite bundle projecting into the apertural area [28]. The latter condition is also present in the cyclostomes *Cinctipora elegans* and *Crisia eburnea* [23–25] that lack a compound tentacle sheath neurite bundle. In phylactolaemates, peripheral areas such as the aperture and body wall are innervated by the diffuse nervous plexus of the tentacle sheath that passes along the duplicature bands and vestibular wall [21].

The diaphragmatic sphincter is innervated by two lateral neurite bundles in most cheilostomes, which in *Electra posidoniae* branch to form a ring-like innervation as also previously reported for *E. pilosa* [42]. Such an innervation has only been found in *Electra* and could represent an apomorphic feature of the genus or closely related species. There is no distinct information on diaphragm innervation on ctenostome bryozoans, but the similar architecture of the peripheral innervation suggests a similar pattern. Nonetheless, this area requires additional attention in future studies for reconstructing a generalized gymnolaemate scheme. From the little data available, cyclostomes have circular neurite bundles concentrated in the diaphragmatic sphincter and are probably connected to the tentacle sheath and vestibular wall innervation [23, 24, 35], which in general was concluded to be similar to other marine bryozoans [36].

Distally of the diaphragm, cheilostome opercular neurite bundles terminate at the respective operculum occlusor muscles. In *Electra pilosa*, the opercular branches innervate the opercular muscles from the lateral sides and form a circular interconnection at the basal side of the zooid [43], which was not observed in the present study. There are, however, small processes on the opercular neurite bundles of *Electra posidoniae* and *Collarina balzaci* that are topologically similar to the described structures. Since operculum occlusor muscles of cheilostomes are homologous to parieto-vestibular muscles of ctenostomes [44], a homologous branch of the opercular neurite bundle might be present in ctenostomes, but has not been described to date.

Phylactolaemate body wall musculature is innervated by a diffuse plexus. Owing to modifications of the original body wall musculature to few bundles associated with polypide protrusion, the innervation of the body wall is accordingly modified to few bundles [5]. Gymnolaemate parietal muscle innervation occurs by a neurite bundle supplying the operculum occlusor and parts of the frontal wall in the cheilostomes *Electra pilosa*, with the latter termed axial residual branch and which presumably terminates in spines of the cystid [43]. The parietal innervation is similar in the cheilostomes of this study, but the branch terminating in the frontal wall is missing, as similarly described in *Chartella papyracea* [41]. Further support for a single parietal neurite bundle as common feature for gymnolaemates is found in the cheilostome *Membranipora membranacea* [37] and the ctenostomes *Alcyonidium gelatinosum* [40] and *Pherusella* *minima*. [29]. Cyclostomes have annular muscles in the lining of the membranous sac innervated by a fine net of diagonal and circular neurite bundles [23, 24]. A set of longitudinal neurite bundles is associated with the distal area of the body wall and probably correlates to longitudinal muscle fibers that are found in few cyclostomes [24] and that appear to have no comparable structure among other bryozoans. Strong modifications of body wall musculature and innervation renders any ground pattern reconstruction difficult and highly speculative.

A plexus innervation of the body wall, termed Hiller’s plexus, was first reported in the cheilostomes *Electra pilosa* [39, 45] and *Membranipora membranacea* [45] and subsequently in the ctenostomes *Alcyonidium polyoum* [46] and *Amathia (Bowerbankia) gracilis* [47]. The plexus originates from two separate neurite bundles that emanate from the proximal basal area of the cerebral ganglion, close to the direct tentacle sheath neurite bundles [39, 48]. It is currently the only known interzooidal nervous connection and associated with interzooidal pores of adjacent zooids [35]. It thus represents an important element in communication of autozooids with heterozooids. None of the more recent studies employing ICC analyses on the nervous system, however, was able to verify Hiller’s plexus so far, which also is the case of the present study.

### Tentacle innervation

In all bryozoan taxa, each tentacle is innervated by four neurite bundles, one medio-frontal, two latero-frontal and one abfrontal [see 26–29, 44 for ctenostomes, 23–25 for cyclostomes, see also 4, 5, 33]. Two additional subperitoneal neurite bundles were described for the cheilostome *Cryptosula pallasiana* [49], but remain questionable as the nature of these subperitoneal cells needs further investigation [4, 5, 33]. In phylactolaemates and the cyclostome *Crisia eburnea*, an additional pair of lateroabfrontal neurite bundles is present yielding in a total number of six epidermal neurite bundles (see references above). In the latter species these bundles do not extend over the full length of the tentacle and terminate in sensory terminations [25].

In most myolaemates, two small rootlets emerge from the CON or the cerebral ganglion that fuse medially to form the medio-frontal neurite bundle [see 26–29, 44 for ctenostomes, 23–25 for cyclostomes]. The current study confirms an identical pattern for cheilostomes. Only in the cyclostome *Cinctipora elegans,* a single root emerges to form the medio-frontal bundle, which, however, is restricted to the lophophoral base [23]. More than two rootlets are present among phylactolaemates and originate either from the CON, the cerebral ganglion or the intertentacular radial nerves [4, 20–22].

The latero-frontal and abfrontal neurite bundles always emerge from the radial nerves, which emanate directly from the circumoral nerve ring and the cerebral ganglion [this study, see references above for other taxa, and 4]. Contrary to the present study, the abfrontal neurite bundle was reported to originate directly from the CON in *Electra pilosa* [42]. However, the current findings in *E. posidoniae* support a situation as in all remaining bryozoans. Only the cyclostome *Cinctipora elegans* shows variation in the origin of the abfrontal tentacle neurite bundle as it can branch off directly or from an intertentacular area [23].

Potential sensory cells on the tentacle tips in connection with the abfrontal neurite bundle were found in the cheilostomes *Electra pilosa, Membranipora membranacea* [50] and *Bicellariella ciliata* [31] and the ctenostome *Flustrellidra hispida* [38]. These were not detected with the applied methods in the current study.

The aforementioned radial nerves are present in all bryozoan taxa and originate from an intertentacular site [4, 5]. All studied gymnolaemates have intertentacular pits containing distinct perikarya [see 32]. According to their cytoskeleton and the bearing of microvilli at a swollen ending, these were regarded as strain receptors in *Electra pilosa* and *Membranipora membranacea* [50].

Structures corresponding to the intertentacular perikarya are also present in non-gymnolaemates. In the cyclostome *Cinctipora elegans* topologically identical intertentacular bases are present that, however, are solid and seem to lack any cavity [23]. In phylactolaemates, intertentacular perikarya are present, but are not associated with any specific structure such as a pit in myolaemates [4].

## Conclusion

This study provides first comprehensive modern data on the nervous system of the largest and still least investigated bryozoan taxon. It also is the first to survey numerous taxa across distant lineages. In general, the nervous system is very uniform among cheilostomes showing only slight variations in some details and is similar to other gymnolaemates. Among other morphological characters, the high similarity of gymnolaemate nervous system supports the sister group relationship to cyclostome bryozoans. In contrast, myolaemates show major differences to phylactolaemates such as the structure of the cerebral ganglion, plexus-like innervation of the tentacle sheath and body wall, and tentacular innervation.

Plexus-like interzooidal nervous connections were not encountered in the current study, but particularly in association with non-feeding heterozooids presumably play an important role. Its exploration remains an important future aspect of nervous system studies in order to obtain a better understanding of coordination and communication particularly in cheilostome bryozoans.

## Data Availability

Datasets are available from the corresponding author upon reasonable request.

## Abbreviations

a: Anus
abr: Annular branch of the trifid neurite bundle
afc: Abfrontal cilia
afn: Abfrontal tentacle neurite bundle
anc: Ancestrula
ca: Cardia
cae: Caecum
caec: Caecum cilia
cg: Cerebral ganglion
cl: Caecum ligament
CON: Circumoral nerve ring
ctsn: Compound tentacle sheath nerve
d: Distal
db: Duplicature bands
dtsn: Direct tentacle sheath nerve
eso: Esophagus
fc: Frontal cilia
inc: Intestinal cilia
int: Intestine
itn: Intertentacular neurite
itp: Intertentacular pit
izp: Interzooidal pores
lb.: Lophophoral base
lc: Lateral cilia
lfc: Laterofrontal cilia
lfn: Laterofrontal tentacle neurite bundle
lvn: Laterovisceral neurite bundle
mfn: Mediofrontal tentacle neurite bundle
mlvn: Mediolaterovisceral neurite bundle
mvn: Mediovisceral neurite bundle
o: Operculum
oc: Ovicell
opn: Opercular nerve
opo: Operculum occlusor
p: Proximal
pbs: Parietal branching site
pdi: Parietodiaphragmatic muscle
pdin: Parietodiaphragmatic nerve
ph: Pharynx
phc: Pharyngeal cilia
php: Pharyngeal nerve plexus
phm: Pharyngeal musculature
pm: Parietal muscle
pmn: Parietal muscle nerve
py: Pylorus
rdn: Radial nerve
rm: Retractor muscle
sph: Sphincter muscle
spn: Sphincter nerve
tc: Tentacle cilia
tco: Tentacle coelom
tfd: Trifid neurite bundle
tm: Tentacle muscle
ts: Tentacle sheath
z: Zooid
